# Evaluation of the Binding Relationship of the RdRp Enzyme to Novel Thiazole/Acid Hydrazone Hybrids Obtainable through Green Synthetic Procedure

**DOI:** 10.3390/polym14153160

**Published:** 2022-08-03

**Authors:** Jehan Y. Al-Humaidi, Mohamed G. Badrey, Ashraf A. Aly, AbdElAziz A. Nayl, Mohie E. M. Zayed, Ohoud A. Jefri, Sobhi M. Gomha

**Affiliations:** 1 Department of Chemistry, College of Science, Princess Nourah bint Abdulrahman University, P.O. BOX 84428, Riyadh 11671, Saudi Arabia; jyalhamidi@pnu.edu.sa; 2 Chemistry Department, Faculty of Science, Fayoum University, El-Fayoum 63514, Egypt; mgb00@fayoum.edu.eg; 3 Chemistry Department, Faculty of Science and Arts-Almandaq, Al-Baha University, Al-Baha 65515, Saudia Arabia; 4 Chemistry Department, Faculty of Science, Organic Division, Minia University, El-Minia 61519, Egypt; ashrafaly63@yahoo.com; 5 Department of Chemistry, College of Science, Jouf University, Sakaka 72341, Saudi Arabia; 6 Department of Chemistry, Faculty of Science, King Abdulaziz University, Jeddah 21589, Saudi Arabia; mzayed@kau.edu.sa (M.E.M.Z.); jeefrio0@gmail.com (O.A.J.); 7 Department of Chemistry, Faculty of Science, Cairo University, Giza 12613, Egypt; 8 Department of Chemistry, Faculty of Science, Islamic University of Madinah, Madinah 42351, Saudi Arabia

**Keywords:** acid hydrazide, hydrazones, grinding, pyrazoles, docking, RdRp enzyme

## Abstract

The viral RNA-dependent RNA polymerase (RdRp) complex is used by SARS-CoV-2 for genome replication and transcription, making RdRp an interesting target for developing the antiviral treatment. Hence the current work is concerned with the green synthesis, characterization and docking study with the RdRp enzyme of the series of novel and diverse hydrazones and pyrazoles. 4-Methyl-2-(2-(1-phenylethylidene)hydrazineyl)thiazole-5-carbohydrazide was prepared and then condensed with different carbonyl compounds (aldehydes and ketones either carbocyclic aromatic or heterocyclic) afforded the corresponding hydrazide-hydrazones. The combination of the acid hydrazide with bifunctional reagents such as acetylacetone, *β*-ketoesters (ethyl acetoacetate and ethyl benzoylacetate) resulted in the formation of pyrazole derivatives. The synthesized compounds were all obtained through grinding method using drops of AcOH. Various analytical and spectral analyses were used to determine the structures of the prepared compounds. Molecular Operating Environment (MOE^®^) version 2014.09 was used to estimate interactions between the prepared thiazole/hydrazone hybrids and RdRp obtained from the protein data bank (PDB: 7bv2) using enzyme-ligand docking for all synthesized derivatives and Remdesivir as a reference. Docking results with the RdRp enzyme revealed that the majority of the investigated drugs bind well to the enzyme via various types of interactions in comparison with the reference drug.

## 1. Introduction

Green synthesis is a developing field in many combined fields of biotechnology that has numerous economic and environmental benefits. Green synthesis has several drawbacks, such as a time-consuming process and repeatability, but it is critical to avoid the generation of undesired or dangerous byproducts through the development of reliable, sustainable, and eco-friendly synthesis techniques [1,2,3,4,5,6,7,8]. Green chemistry has defined and well-established principles such as the prevention of wastage, optimizing both atom economy and energy efficiency, developing less dangerous chemical synthesis, developing less dangerous products, avoiding chemical derivatives, employing cleaner reaction conditions, avoiding pollution, using renewable feed stocks, using non-stoichiometric reagents, designing chemicals that degrade after use, and minimizing the possibility for accidents. Green organic synthesis can be used to build synthetic techniques for biologically active and industrially valuable chemicals [9,10,11].

1,3-Thiazole derivatives were considered extensively in generating novel lead compounds and drug development. Thiazole derivatives derived from thiosemicarbazones are scaffolds of many synthetic, natural, and semi-synthetic drugs that show a wide range of pharmacological activities, including anticancer, anti-inflammatory, and antiparasitic activities [2,6,12,13,14,15,16,17,18,19]. On the other hand, acid hydrazides were extensively utilized as building blocks in a variety of synthetic reactions, including 1,3,4-thiadiazoles, 1,3,4-oxadiazoles, 1,2,4-triazoles, 1,2,4,5-tetrazines, and others [20]. Hydrazides are highly bioactive chemicals with broad biological activities, including glycogen phosphorylase inhibitors, insecticides, HIV inhibitors, myeloperoxidase inhibitors, and antituberculous medications (isoniazid) [21]. Furthermore, hydrazide-hydrazones are commonly utilized as enzyme inhibitors [22]. Some hydrazones, for example, have been examined as a new class of dual inhibitors of thymidine phosphorylase and cancer cell growth, which merits further investigation for anti-cancer drug development [23,24]. Recently, the direct synthesis of hydrazides from their acids was reported utilizing microwave irradiation without using any toxic solvents and other ancillary reagents such as surfactants, as well as its validation using green chemistry principles [25,26].

Molecular docking investigations are the most needed and effective discipline to the rational design of new therapeutic materials to treat the human diseases. Due to the important roles of enzymes groups for drug targets, the identification of possible ligand-enzyme interactions plays vital roles in much drug discovery research. SARS-CoV-2 is a single-stranded positive-sense RNA virus with a 30 kb genome arranged in 14 open reading frames [27,28]. All genome replication and gene transcription are reliant on a binding protein known as RdRp (RNA-dependent RNA polymerase). The latter comprises a catalytic component nsp12 (non-structural protein 12) as well as two auxiliary subunits (nsp7 and nsp8) [29,30]. Anti-coronavirus drug development has focused on RdRp as the most attractive target, due to its important role and the paucity of homologues in human beings [31,32]. Predicting ligands that bind with adequate strength to a corresponding protein is a difficult task in biochemistry that is closely tied to the identification of new drug candidates.

## 2. Results and Discussion

### 2.1. Chemistry

**Scheme 1:**
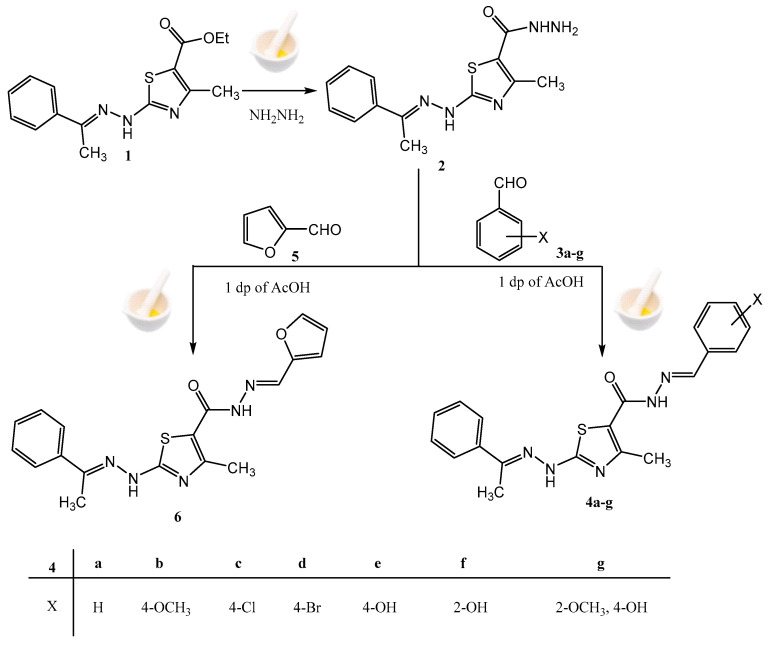
Synthesis of acid hydrazones **4a**–**g** and **6**.

The thiazole ester derivative **1** [33] is used as a key precursor for synthesizing new compounds using simple and green procedures. The conversion of the ester **1** into the corresponding acid hydrazide **2** is performed through the reaction with hydrazine hydrate using grinding method in mortar with a few drops of AcOH (Scheme 1). The conversion of the ester group into the hydrazide functionality is strongly assisted from the spectral data (^1^H-NMR and IR); in the IR spectrum, the amide carbonyl group absorption band is shown at 1669 cm^−1^; in addition, the amino and NH functions showed bands at 3230, 3346 and 3419 cm^−1^. The ^1^H-NMR (in DMSO-*d*6) spectra illustrated no ethoxy pattern signals exhibited by the starting ester; the hydrazide protons displayed signals at 5.02 ppm (NH_2_) and 11.23 ppm (NH) in addition to the expected signals (see Experimental section).

Carbonyl compounds (aldehydes and ketones), which are major constituents of food aroma and widely distributed in the environment, can be used to condensate with the acid hydrazide **2** to afford the corresponding hydrazones under grinding method with few drops of AcOH. Thus, the reaction of the acid hydrazide **2** with aromatic aldehydes either carbocyclic (**3a**–**g**) or heterocyclic **5** gives the corresponding hydrazones **4a**–**g** and **6**, respectively (Scheme 1).

The ^1^H-NMR spectra of the condensation products, hydrazide-hydrazones **4a**–**g** and **6**, all lack the signal attributed to NH_2_ groups, indicating the hydrazide-hydrazone formation; in addition, the imine proton CH=N- is clearly apparent in the range 7.92–8.06 ppm. In the ^13^C-NMR of the derivative **4d**, an apparent signal at 162.7 ppm corresponds to the amide group carbon signal.

**Scheme 2:**
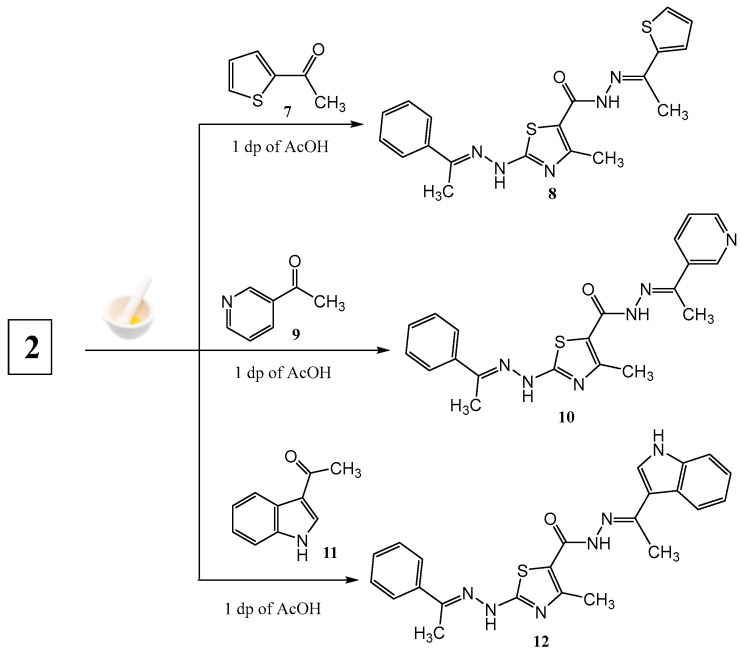
Synthesis of acid hydrazones **8**, **10** and **12**.

The hydrazone series is extended by the reaction of acid hydrazide **2** with heterocyclic ketones **7**, **9**, and **11** using grinding method to furnish compounds **8**, **10**, and **12**, respectively; the synthesis is easy and efficient as nothing is added except for starting materials and drops of AcOH affected by mechanical power (Scheme 2).

The spectral data obtained for these compounds are in accordance with the expected structures. The ^1^H-NMR spectrum of compound **8** showed three singlet signals at 2.31, 2.40 and 2.45 ppm for the three methyl groups. In addition, the aromatic region displays signals from 6.63–7.79 ppm integrating for 8 protons. The ^13^C-NMR of compound **12** displayed three signals at 14.9, 17.8, and 20.9 ppm related to the three methyl groups carbon, and also a signal at 162.4 ppm is attributable to the amide carbon.

**Scheme 3:**
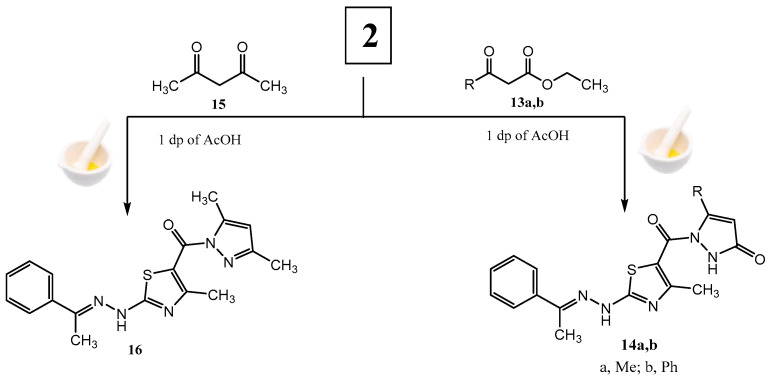
Synthesis of pyrazole derivatives **14a**,**b** and **16**.

Heterocyclization of the key intermediate acid hydrazide **2** can be accomplished through its condensation with β-ketoesters **13a**,**b** namely ethyl acetaoacetate and ethyl benzoylacetate as bifunctional reagents, which indeed provide a rich opportunity for heterocycles annulation. Grinding the reactants with a few drops of AcOH leads to the biologically valuable pyrazolone derivatives **14a**,**b** in qualitative yields (Scheme 3). The structures of these compounds are confirmed from their spectral results; thus, in the ^1^H-NMR of the pyrazolone **14b**, the appearance of multiplet signals in the range 7.19–8.25 with integral intensity corresponding to eleven protons (aromatic and pyrazolone-4H) indicates the incorporation of an additional phenyl group from ethyl benzoylacetate. Furthermore, mass spectrum gives the correct molecular ion band at m/e = 417.

Additionally, the condensation of acid hydrazide **2** with other bifunctional reagents as β-diketones as acetylacetone **15** under same conditions furnishes the dimethylpyrazole derivative **16**.

### 2.2. Molecular Docking Studies

**Figure 1:**
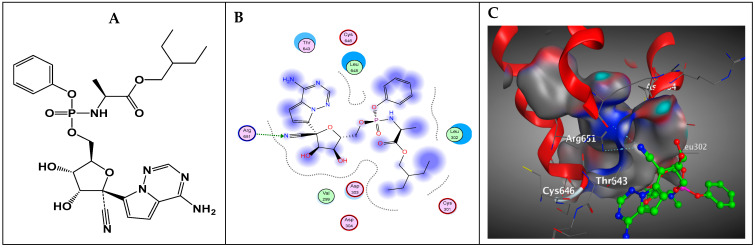
(**A**) Structure of the standard inhibitor Remedesivir, (**B**) 2D pose and (**C**) surface map of the interaction of Remedisvir into the active site of SARS-Cov-2 RdRp (PDB: 7bv2).

Enzyme-ligand docking investigations were done for all derivatives **1**, **2**, **4a**–**g**, **6**, **8**, **10**, **12**, **14a**, **14b**, **16** and *Remdesivir* as a well-known antiviral drug aiming to estimate the interactions of the prepared thiazole/hydrazone hybrids with RdRp produced from the protein data bank (PDB: 7bv2) using MOE^®^ version 2014.09. The docking process was confirmed by redocking the co-crystallised RdRp inhibitor *Remedisivir* with data obtained from *Remdesivir* [34] (Figure 1).

**Figure 2:**
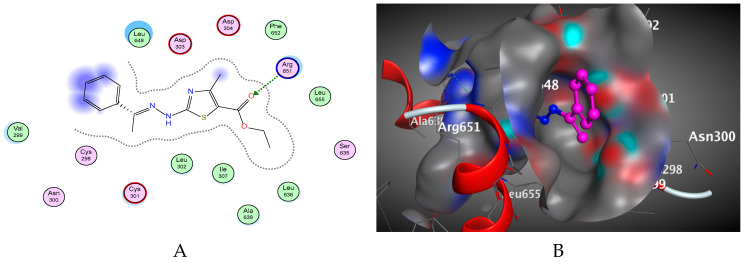
(**A**) 2D pose and (**B**) surface map of the interaction of **1** into the active sites of SARS-CoV-2 RdRp (PDB: 7bv2).

**Figure 3:**
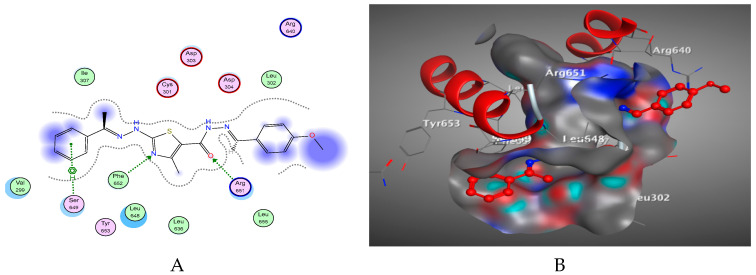
(**A**) 2D pose and (**B**) surface map of the interaction of **4b** into the active sites of SARS-CoV-2 RdRp (PDB: 7bv2).

**Figure 4:**
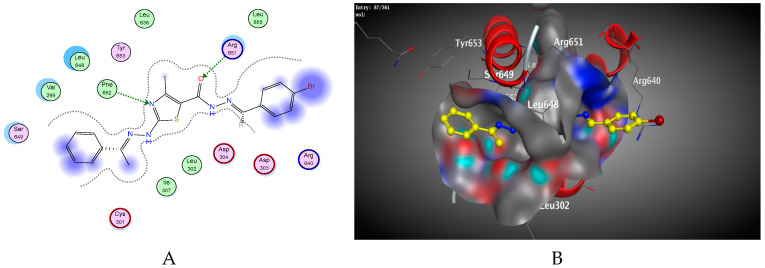
(**A**) 2D pose and (**B**) surface map of the interaction of **6** into the active sites of SARS-CoV-2 RdRp (PDB: 7bv2).

**Figure 5:**
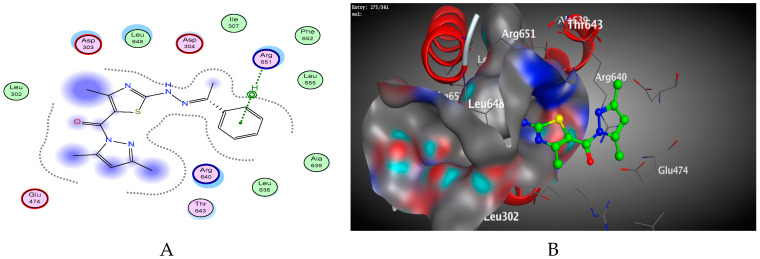
(**A**) 2D pose and (**B**) surface map of the interaction of **16** into the active sites of SARS-CoV-2 RdRp (PDB: 7bv2).

All hybrids have been already docked into the active site of RdRp, and the binding free energy (ΔG) from the main docked poses was shown in Table 1. Figure 2, Figure 3, Figure 4 and Figure 5 depict the most favorable poses of the compounds investigated. Most of these examined materials have potential activity against the SARS-CoV-2 RdRp enzyme as the energy scores ranging from −4.52 to −6.63, while the ΔG is ranging from −0.6 to −3.2 Kcal/mol in comparison to the value from –2.5 to −5.1 Kcal/mol for the *Remdesivir* reference drug.

Low Ki values imply great potency, and a molecule must fall within the micromolar range to be regarded as a lead chemical or hit. Compounds **1** and **6** are the most potent of the other examined compounds since they require the lowest Ki values of 2.61 × 10^−5^ and 1.4 × 10^−5^ µM to be classified as drugs.

The docking results of the Remedisvir reference drug are completely consistent with the mode of actions of thiazole/hydrazone derivatives. Moreover, the reference drug was stabilized within the active sites via a critical two H-bonding donor and acceptor with the amino acid residues, ARG651. The docking results with RdRp enzyme indicated that most of the target hybrids showed significant interactions with the enzyme and displayed many interactions in comparison to the Remedisvir drug. Compound **2** revealed an interaction typical to Remedisvir with two strong hydrogen bonding donor and acceptor with ARG651 amino acid residues.

All the tested compounds exhibited interactions with the conserved amino acid ARG651. Although all the tested derivatives showed extra interaction, compounds **2** and **16** lacked any extra binding other than with ARG651 amino acid residue.

Only **4a**–**g** and **8** derivatives possess extra H-acceptor bonding interaction with PHE652; however, compounds **4a**, **4b**, **4f**, **4g**, **8**, and **12** possess hydrophobic pi-H interaction with SER649 amino acid residue (see compound **4b**, Figure 3).

Meanwhile, the amino acid residue ASP304 was obviously interacted with only **4f** and **6** derivatives through H-donor and H-acceptor binding, respectively (Table 1) (see compound **6**, Figure 4).

The current docking results follow these prior data, indicating the viability of utilizing these medications in the global fight against COVID-19. Furthermore, the favorable safety profile of thiazole/hydrazone supports additional steps into in vitro research to confirm anti-SARS-CoV-2 potential.

### 2.3. Physicochemical Characteristics, Pharmacokinetics, and Drug-Likeness Profile; In Silico Prediction

The design and selection of novel drugs could be considered as a complicated issue, whereas estimating the pharmacokinetic characteristics of novel drugs is a critical phase in the drug development process that can directly lead to optimizing the efforts to recover analogs [35]. Unfortunately, ADME parameters (distribution, excretion, absorption and metabolism) revealed improper property, in addition to the needed costs to develop novel drugs (Other information about physicochemical characteristics, pharmacokinetics, drug-likeness profile and in silico prediction was explained in Supplementary Materials [36,37,38,39,40]).

Table 2 showed the data obtained for the tested compounds **1**, **2**, **4a**–**g**, **6**, **8**, **10**, **12**, **14a**, **14b**, and **16** following the Lipinski’s rule with Log P values of 1.92–4.46 (<5), MW values of 289.36–456.36 (<500), HBD with a range of 1–3 (≤5), and HBA with a range of 4–5 (<10). They will theoretically demonstrate strong oral absorption and these characteristics cannot be due to differences in their bioactivities. Moreover, the values of topological PSA for the rings of the compounds ranged from 91.82 to 135.22 A2 (<140 A2) and the corresponding percentages for oral absorption ranged 77.35–62.35%, showing considerable permeability, absorption and transport across biological membranes. Moreover, solubility and the drug-likeness models scores of target materials were ensured by Molsoft software as shown in Table 3. It is worth mentioning that aquatic solubility can modify the absorption and distribution properties. The more positive the drug-likeness model’s scores were, the more likely it was drug molecules. The LogS values of the target compounds range from 1.15–7.96 mg/L, and the most positive model score for compounds **4e** and **4f**.

The results for ADME parameters are illustrated in Table 4. The results from prepared materials with moderate CNS absorption range from 0.02 to 0.44 (≤0.1). The examined materials showed medium to low cell permeability in the Caco-2 and MDCK models ranging from 0.46 to 22.52 and 0.03 to 90.63 nm/s, respectively.

This is consistent with the non-CYP2D6 enzyme and therefore can pretend to have no interaction with the CYP2D6 inhibitor and/or inducers. Moreover, all prepared materials **1**, **2**, **4a**–**g**, **6**, **8**, **10**, **12**, **14a**, **14b**, and **16** illustrated considerable human intestinal absorption values from 62.35 to 77.02%. This indicates well-absorbed compounds especially for **4c**, which showed 77.02% intestinal absorption. The compounds studied were found to be considerable with regard to bonding to human plasma proteins from 74.99 to 94.04%, except for **2** which was as low as 56.98% bonding for plasma protein.

## 3. Experimental Section

### 3.1. Chemistry

Unless otherwise specified, all of the chemicals and solvents are of commercial grade and are utilized without additional purification. Using Merck silica gel GF254 plates (Merck, Darmstadt Germany), analytical thin-layer chromatography (TLC) was carried out. The melting points of the products were measured on an X-4 melting point apparatus (Bibby Sci. Lim. Stone, Staordshire, UK). The IR spectra were recorded with a Nicolet Nexus 670 FT-IR spectrometer (Nicolet Madison, WI, USA). The ^1^H-NMR spectra were recorded using Bruker Avance (Bruker Biospin, Karlsruhe, Germany) 500 Spectrometer in DMSO-*d*_6_. Chemical shifts (*δ*) are given in ppm relative to tetramethylsilane as the internal reference, with coupling constants (*J*) in Hz. The ^13^C-NMR spectra were recorded at 125 MHz. Elemental analyses were measured using EuroEA elemental analyzer instrument (GmbH & Co.KG, Hanau, Germany). The safety measures required for the experimental technique were taken into account, including respiratory protection, gloves, and the wearing of a face shield (with safety glasses or goggles to protect against volatile and harmful particles). Additionally, the experiment is being conducted within the fume hood.

**Synthesis of 4-methyl-2-(2-(1-phenylethylidene)hydrazineyl)thiazole-5-carbohydrazide (2).**

Two drops of AcOH were mixed with a mixture of thiazole ester **1** (10 mmol, 3.03 g) and the hydrazine hydrate (15 mmol) and then the mixture was ground with a pestle in a mortar at 25 °C. During the course of the reaction, the mixture became a sticky paste that finally solidified for 20 min on completion. TLC (EtOAc: n-hexane 1:1) was used to monitor the reaction progress. The reaction mixture was poured into water, filtered, and finally the crude product was purified by recrystallization from EtOH to give the acid hydrazide **2** as white solid (76%); mp 197–199 °C; ^1^H-NMR (500 MHz, DMSO-*d*_6_) *δ* 11.72 (s, 1H, NH), 11.23 (s, 1H, NH), 5.02 (s, 2H, NH_2_), 7.61–7.25 (m, 5H, Ar-H), 2.43 (s, 3H, CH_3_), 2.27 (s, 3H, CH_3_) ppm; IR (KBr): *v* 1669, 1703 (2C=O), 2931, 3148 (C-H), 3230, 3346, 3419 (NH and NH_2_) cm^−1^; MS m/z (%): 289 (M^+^, 28). Anal. calcd for C_13_H_15_N_5_OS (289.36): C, 53.96; H, 5.23; N, 24.20. Found: C, 53.73; H, 5.17; N, 24.05.

**General procedure for the synthesis of acid hydrazones 4a**–**g**, **6**, **8**, **10 and 12.**

Two drops of AcOH were added into a mixture of acid hydrazide derivative **2** (1 mmol, 0.289 g) and the appropriate substituted aldehyde **3a**–**g**, **5**, or ketone, namely 2-acetylthiophene **7**, 2-acetylpyridine **9b**, or 3-acetylindole **11** (1 mmol for each) and then the mixture was ground with a pestle in a mortar at 25 °C. During the course of the reaction, the mixture became a sticky paste which finally solidified for 15–25 min on completion. TLC (EtOAc: *n*-hexane 1:1) was used to monitor the reaction progress. The reaction mixture was emptied into water, filtered, and the crude products were purified by recrystallization from the suitable solvent to obtain products **4a**–**g**, **6**, **8**, **10**, or **12**. The following are the spectral data and physical constants for the products:

**N′-(Benzylidene)-4-methyl-2-(2-(1-phenylethylidene)hydrazineyl)thiazole-5-carbohydrazide (4a).** White solid (74%); m.p. 160–162 °C (EtOH); ^1^H-NMR (500 MHz, DMSO-*d*_6_) *δ* 12.23 (s, 1H, NH), 11.46 (s, 1H, NH), 8.06 (s, 1H, CH=N), 7.64–7.21 (m, 10H, Ar-H), 2.44 (s, 3H, CH_3_), 2.23 (s, 3H, CH_3_) ppm; IR (KBr): *v* 1590 (C=N), 1685 (C=O), 2885, 2920 (C-H), 3205, 3440 (2N-H) cm^−1^; MS *m/z* (%): 377 (M^+^, 29). Anal. Calcd. For C_20_H_19_N_5_OS (377.47): C, 63.64; H, 5.07; N, 18.55. Found C, 63.52; H, 5.01; N, 18.38%.

**N′-(4-Methoxybenzylidene)-4-methyl-2-(2-(1-phenylethylidene)hydrazineyl)thiazole-5-carbohydrazide (4b).** Yellow solid (76%); m.p. 181–183 °C (EtOH); ^1^H-NMR (500 MHz, DMSO-*d*_6_) *δ* 12.14 (s, 1H, NH), 11.21 (s, 1H, NH), 8.00 (s, 1H, CH=N), 7.60–6.96 (m, 9H, Ar-H), 3.81 (s, 3H, OCH_3_), 2.43 (s, 3H, CH_3_), 2.16 (s, 3H, CH_3_) ppm; ^13^C-NMR (DMSO-*d*_6_) *δ* 14.9, 17.6 (2CH_3_), 55.8 (OCH_3_), 114.6, 114.9, 117.6, 117.8, 127.1, 128.8, 129.0, 134.0, 139.7, 142.5, 142.9, 145.0, 161.3, 162.6 (Ar-C, C=N, C=O) ppm; IR (KBr): *v* 1600 (C=N), 1690 (C=O), 2920, 2960 (C-H), 3205, 3400 (2N-H) cm^−1^; MS *m/z* (%): 407 (M^+^, 52). Anal. Calcd. For C_21_H_21_N_5_O_2_S (407.49): C, 61.90; H, 5.19; N, 17.19. Found C, 61.90; H, 5.19; N, 17.19%.

**N′-(4-Chlorobenzylidene)-4-methyl-2-(2-(1-phenylethylidene)hydrazineyl)thiazole-5-carbohydrazide (4c).** Yellow solid (78%); m.p. 201–203 °C (DMF); ^1^H-NMR (500 MHz, DMSO-*d*_6_) *δ* 12.37 (s, 1H, NH), 11.15 (s, 1H, NH), 8.03 (s, 1H, CH=N), 7.65–7.46 (m, 9H, Ar-H), 2.43 (s, 3H, CH_3_), 2.16 (s, 3H, CH_3_) ppm; IR (KBr): *v* 1570 (C=N), 1680 (C=O), 2880, 2925 (C-H), 3190, 3360, (2N-H) cm^−1^; MS *m/z* (%): 411 (M^+^, 82). Anal. Calcd. For C_20_H_18_ClN_5_OS (411.91): C, 58.32; H, 4.40; N, 17.00. Found C, 58.26; H, 4.35; N, 16.81%.

**N′-(4-Bromobenzylidene)-4-methyl-2-(2-(1-phenylethylidene)hydrazineyl)thiazole-5-carbohydrazide (4d).** Yellow solid (77%); m.p. 193–194 °C (DMF); ^1^H-NMR (500 MHz, DMSO-*d*_6_) *δ* 11.81 (s, 1H, NH), 10.31 (s, 1H, NH), 8.03 (s, 1H, CH=N), 7.70–7.15 (m, 9H, Ar-H), 2.43 (s, 3H, CH_3_), 2.18 (s, 3H, CH_3_) ppm; IR (KBr): *v* 1575 (C=N), 1687 (C=O), 2890, 2930 (C-H), 3200, 3375 (2N-H) cm^−1^; ^13^C-NMR (DMSO-*d*_6_) *δ* 14.9, 17.7 (2CH_3_), 115.2, 119.0, 121.3, 124.3, 126.0, 126.8, 129.5, 131.7, 133.9, 140.6, 144.6, 149.62, 157.0, 162.7 (Ar-C, C=N, C=O) ppm; MS *m/z* (%): 356 (M^+^, 39). Anal. Calcd. For C_20_H_18_BrN_5_OS (456.36): C, 52.64; H, 3.98; N, 15.35. Found C, 52.52; H, 3.77; N, 15.19%.

**N′-(2-Hydroxybenzylidene)-4-methyl-2-(2-(1-phenylethylidene)hydrazineyl)thiazole-5-carbohydrazide (4e).** Creamy solid (82%); m.p. 155–157 °C (EtOH); ^1^H-NMR (500 MHz, DMSO-*d*_6_) *δ* 11.84 (s, 1H, NH), 10.06 (s, 1H, NH), 8.50 (s, 1H, OH), 8.04 (s, 1H, CH=N), 7.62–6.82 (m, 9H, Ar-H), 2.42 (s, 3H, CH_3_), 2.22 (s, 3H, CH_3_) ppm; ^13^C-NMR (DMSO-*d*6) *δ* 14.7, 18.0 (2CH_3_), 108.3, 116.7, 120.0, 120.1, 127.7, 131.6, 131.8, 136.4, 138.7, 139.6, 140.2, 145.0, 153.0, 156.7, 157.1, 162.2 (Ar-C, C=N, C=O) ppm; IR (KBr): *v* 1600 (C=N), 1684 (C=O), 2885, 2960 (C-H), 3220, 3410 (2N-H) cm^−1^; MS *m/z* (%): 393 (M^+^, 63). Anal. Calcd. For C_20_H_19_N_5_O_2_S (393.13): C, 61.05; H, 4.87; N, 17.80. Found C, 61.01; H, 4.74; N, 17.71%.

**N′-(4-Hydroxybenzylidene)-4-methyl-2-(2-(1-phenylethylidene)hydrazineyl)thiazole-5-carbohydrazide (4f).** White solid (81%); m.p. 171–173 °C (EtOH); ^1^H-NMR (500 MHz, DMSO-*d*_6_) *δ* 11.20 (s, 1H, NH), 10.80 (s, 1H, NH), 9.75 (s, 1H, OH), 7.98 (s, 1H, CH=N), 7.53–6.73 (m, 9H, Ar-H), 2.42 (s, 3H, CH_3_), 2.22 (s, 3H, CH_3_) ppm; IR (KBr): *v* 1605 (C=N), 1688 (C=O), 2880, 2925 (C-H), 3320, (br, N-H) cm^−1^; MS *m/z* (%): 393 (M^+^, 19). Anal. Calcd. For C_20_H_19_N_5_O_2_S (393.47): C, 61.05; H, 4.87; N, 17.80. Found C, 61.17; H, 4.69; N, 17.64%.

**N′-(4-Hydroxy-2-methoxybenzylidene)-4-methyl-2-(2-(1-phenylethylidene)hydrazineyl) thiazole-5-carbohydrazide (4g).** Yellow solid (80%); m.p. 181–183 °C (EtOH); ^1^H-NMR (500 MHz, DMSO-*d*_6_) *δ* 12.31 (s, 1H, NH), 10.39 (s, 1H, NH), 9.54 (s, 1H, OH), 7.94 (s, 1H, CH=N), 7.47–6.78 (m, 8H, Ar-H), 3.79 (s,3H, OCH_3_) 2.43 (s, 3H, CH_3_), 2.25 (s, 1H, CH_3_) ppm; ^13^C-NMR (DMSO-*d*_6_) *δ* 14.9, 17.8 (2CH_3_), 56.4 (OCH_3_), 110.2, 116.2, 121.5, 125.9, 126.4, 128.9, 129.6, 135.1, 137.0, 139.9, 140.2, 145.6, 148.5, 149.4, 159.54, 162.5 (Ar-C, C=N, C=O) ppm; IR (KBr): *v* 1595 (C=N), 1690 (C=O), 2890, 2930 (C-H), 3120, 3200 (N-H) cm^−1^; MS *m/z* (%): 349 (M^+^, 36). Anal. Calcd. For C_21_H_21_N_5_O_3_S (423.49): C, 59.56; H, 5.00; N, 16.54. Found C, 59.42; H, 4.92; N, 16.41%.

**N′-(Furan-2-ylmethylene)-4-methyl-2-(2-(1-phenylethylidene)hydrazineyl)thiazole-5-carbohydrazide (6).** Yellow solid (76%); m.p. 188–190 °C (EtOH); ^1^H-NMR (500 MHz, DMSO-*d*_6_) *δ* 12.35 (s, 1H, NH), 11.32 (s, 1H, NH), 7.92 (s, 1H, CH=N), 7.79–6.59 (m, 8H, Ar-H), 2.42 (s, 3H, CH_3_), 2.37 (s, 3H, CH_3_) ppm; IR (KBr): *v* 1590 (C=N), 1685 (C=O), 2960 (C-H), 3208, 3410 (N-H) cm^−1^; MS *m/z* (%): 387 (M^+^, 100). Anal. Calcd. For C_18_H_17_N_5_O_2_S (367.43): C, 58.84; H, 4.66; N, 19.06. Found C, 58.79; H, 4.40; N, 19.02%.

**4-Methyl-2-(2-(1-phenylethylidene)hydrazineyl)-N′-(1-(thiophen-2-yl)ethylidene)thiazole-5-carbohydrazide (8).** Yellow solid (80%); m.p. 177–179 °C (DMF); ^1^H-NMR (500 MHz, DMSO-*d*_6_) *δ* 12.27 (s, 1H, NH), 11.12 (s, 1H, NH), 7.79–6.63 (m, 8H, Ar-H), 2.45 (s, 3H, CH_3_), 2.40 (s, 3H, CH_3_), 2.31 (s, 3H, CH_3_) ppm; ^13^C-NMR (DMSO-*d*_6_) *δ* 14.6, 17.5, 21.9 (3CH_3_), 114.6, 119.3, 122.3, 125.4, 125.7, 126.5, 128.0, 132.8, 135.0, 141.4, 142.6, 148.6, 157.5, 162.3 (Ar-C, C=N, C=O) ppm; IR (KBr): *v* 1593 (C=N), 1689 (C=O), 2957 (C-H), 3215, 3406 (N-H) cm^−1^; MS *m/z* (%): 397 (M^+^, 61). Anal. Calcd. For C_19_H_19_N_5_OS_2_ (397.52): C, 57.41; H, 4.82; N, 17.62. Found C, 57.37; H, 4.68; N, 17.44%.

**4-Methyl-2-(2-(1-phenylethylidene)hydrazineyl)-N′-(1-(12yridine-3-yl)ethylidene)thiazole-5-carbohydrazide (10).** Yellow solid (72%); m.p. 191–193 °C (EtOH); ^1^H-NMR (500 MHz, DMSO-*d*_6_) *δ* 11.90 (s, 1H, NH), 11.23 (s, 1H, NH), 8.91–7.41 (m, 9H, Ar-H), 2.42 (s, 3H, CH_3_) 2.30 (s, 3H, CH_3_), 2.21 (s, 3H, CH_3_) ppm; IR (KBr): *v* 1580 (C=N), 1682 (C=O), 2850, 2922 (C-H), 3120, 3190 (N-H) cm^−1^; MS *m*/*z* (%): 392 (M^+^, 71). Anal. Calcd. For C_20_H_20_N_6_OS (392.48): C, 61.21; H, 5.14; N, 21.41. Found C, 61.03; H, 5.06; N, 21.36%.

**N′-(1-(1*H*-Indol-3-yl)ethylidene)-4-methyl-2-(2-(1-phenylethylidene)hydrazineyl)thiazole-5-carbohydrazide (12).** Yellow solid (89%); m.p. 174–176 °C (DMF); ^1^H-NMR (500 MHz, DMSO-*d*_6_) *δ* 11.87 (s, 1H, NH), 11.50 (s, 1H, NH), 10.28 (s, 1H, NH), 8.34–7.18 (m, 10H, Ar-H), 2.46 (s, 3H, CH_3_), 2.40 (s, 3H, CH_3_), 2.32 (s, 3H, CH_3_) ppm; ^13^C-NMR (DMSO-*d*_6_) *δ* 14.9, 17.8, 20.9 (3CH_3_), 112.3, 112.6, 120.9, 121.8, 122.13, 122.8, 122.9, 123.2, 128.9, 134.8, 137.2, 137.7, 142.5, 144.5, 150.7, 152.7, 159.9, 162.4 (Ar-C, C=N, C=O) ppm; IR (KBr): *v* 1565 (C=N), 1685 (C=O), 2850, 2940 (C-H), 3235, 3333 (N-H) cm^−1^; MS *m/z* (%): 430 (M^+^, 100). Anal. Calcd. For C_23_H_22_N_6_OS (430.53): C, 64.17; H, 5.15; N, 19.52. Found C, 64.02; H, 5.27; N, 19.35%.

**General procedure for the synthesis of pyrazoles 14a**, **14b and 16.**

One drop of glacial acetic acid was added into a mixture of acid hydrazide **2** (1 mmol, 0.289) and the appropriate bifunctional reagent, namely ethyl acetoacetate **13a** ethylbenzoylacetate **13b** or acetylacetone **15** (1 mmol for each), and then the mixture was ground with a pestle in a mortar at 25 °C. During the course of the reaction, the mixture became a sticky paste that finally solidified for 20–30 min on completion. The products were collected, washed with EtOH and recrystallized from EtOH to furnish pure compounds **14a**,**b** and **16**, respectively.

**5-Methyl-1-(4-methyl-2-(2-(1-phenylethylidene)hydrazineyl)thiazole-5-carbonyl)-1**,**2-dihydro-3*H*-pyrazol-3-one (14a).** Yellow solid (74%); m.p. 161–163 °C; ^1^H-NMR (500 MHz, DMSO-*d*_6_) *δ* 11.66 (s, 1H, NH), 11.34 (s, 1H, NH), 7.87–7.45 (m, 5H, Ar-H), 7.20 (s, 1H, pyrazole-H4), 2.46 (s, 3H, CH_3_), 2.35 (s, 3H, CH_3_), 2.12 (s, 3H, CH_3_) ppm; ^13^C-NMR (DMSO-*d*_6_) *δ* 14.6, 17.4, 21.8 (3CH_3_), 105.5, 119.7, 124.4, 126.6, 133.8, 139.9, 140.1, 140.2, 154.4, 156.7, 162.5, 166.2 (Ar-C, C=N, C=O) ppm; IR (KBr): *v* 1560 (C=N), 1655, 1695 (2C=O), 2935 (C-H), 3203, 3312 (N-H) cm^−1^; MS *m/z* (%): 355 (M^+^, 36). Anal. Calcd. for C_17_H_17_N_5_O_2_S (355.42): C, 57.45; H, 4.82; N, 19.71. Found C, 57.60; H, 4.69; N, 19.51%.

**1-(4-Methyl-2-(2-(1-phenylethylidene)hydrazineyl)thiazole-5-carbonyl)-5-phenyl-1**,**2-dihydro-3*H*-pyrazol-3-one (14b).** Yellow solid (79%); m.p. 193–195 °C; ^1^H-NMR (500 MHz, DMSO-*d*_6_) *δ* 10.63 (s, 1H, NH), 8.25–7.19 (m, 11H, Ar-H and NH), 6.61 (s, 1H, pyrazole-H4), 2.63 (s, 3H, CH_3_), 2.44 (s, 3H, CH_3_) ppm; ^13^C-NMR (126 MHz, DMSO-*d*_6_) *δ* 14.9, 20.9 (2CH_3_), 106.8, 116.6, 117.7, 121.3, 123,7, 125.9, 129.6, 130.2, 133.5, 137.4, 138.7, 141.3, 149.7, 159.7, 162.1, 167.2 (Ar-C, C=N, C=O) ppm; IR (KBr): *v* 1590 (C=N), 1695 (2C=O), 2855, 2965 (C-H), 3125, 33,415 (N-H) cm^−1^; MS *m/z* (%): 417 (M^+^, 100). Anal. Calcd. for C_22_H_19_N_5_O_2_S (417.49): C, 63.29; H, 4.59; N, 16.78. Found C, 63.14; H, 4.38; N, 16.55%.

**(3**,**5-Dimethyl-1*H*-pyrazol-1-yl)(4-methyl-2-(2-(1-phenylethylidene)hydrazineyl)thiazol-5-yl)methanone (16).** Yellow solid (76%); m.p. 180–182 °C; ^1^H-NMR (500 MHz, DMSO-*d*_6_) *δ* 11.84 (s, 1H, NH), 7.86–7.01 (m, 6H, Ar-H and pyrazole-H4), 2.47 (s, 3H, CH_3_), 2.43 (s, 3H, CH_3_), 2.30 (s, 3H, CH_3_), 2.21 (s, 3H, CH_3_) ppm; IR (KBr): *v* 1595 (C=N), 1692 (C=O), 2872, 2953 (C-H), 3168, 3403 (N-H) cm^−1^; MS *m/z* (%): 353 (M^+^, 48). Anal. Calcd. for C_18_H_19_N_5_OS (353.44): C, 61.17; H, 5.42; N, 19.82. Found C, 61.03; H, 5.28; N, 19.61%.

### 3.2. Docking Study

All information about the docking study was explained in Supplementary Materials.

### 3.3. Target Optimization

All information about target optimization was explained in Supplementary Materials.

### 3.4. Docking of the Target Molecules to SARS-CoV-2 RdRp Active Site

All information about the docking of the target molecules to SARS-CoV-2 RdRp active site was explained in Supplementary Materials.

### 3.5. Physicochemical Properties, Pharmacokinetics, and Drug-Likeness Profile; In Silico Prediction

All information about physicochemical properties, pharmacokinetics, drug-likeness profile and in silico prediction was explained in Supplementary Materials.

## 4. Conclusions

In this research, we designed, evaluated, and predicted the binding interactions between newly synthesized thiazole/hydrazone hybrids and RdRp using enzyme-ligand docking for the prepared compounds. A simple efficient green protocol was used to synthesis hydrazone derivatives **4a**–**h**, **6**, **8**, **10**, **12**, and pyrazole-containing compounds **14a**,**b**, and **15**. All compounds docked successfully into RdRp’s active site. The majority of the investigated compounds showed activity against the SARS-CoV-2 RdRp enzyme, with energy scores ranging from –4.52 to –6.63 and ΔG = –0.6 to 3.2 Kcal/mol, which are similar to the reference Remedisvir (ΔG = –2.5 to –5.1 Kcal/mol). The docking results with the RdRp enzyme revealed that the majority of the compounds examined bind well to the enzyme and have many interactions that are similar to the reference. Compound **2** revealed an interaction typical to Remedisvir with two strong hydrogen bonding donor and acceptor with ARG651 amino acid residues. All the tested compound exhibited interactions with the conserved amino acid ARG651. Although all the tested derivatives showed extra interaction, compounds **2** and **16** lacked any extra binding other than with ARG651 amino acid residue.

## Figures and Tables

**Table 1:** Energy scores and types of interactions observed for the investigated compounds and the reference Remedisvir in the active sites of RdRp (PDB: 7bv2).

Compound	Energy Score	Inhibition Constant *K*i (µM)	ΔG (Kcal/mol) ^a^	Ligand-Receptor Interactions		
				Residue	Type	Length (Å)
**1**	−6.26	2.61 × 10^−5^	−4.5	ARG651	H-acceptor	2.73
**2**	−5.91	4.72 × 10^−5^	−1.2 −5.7	ARG651 ARG651	H-acceptor H-acceptor	3.02 2.84
**4a**	−5.30	1.32 × 10^−4^	−2.8 −0.9 −0.6	ARG651 PHE652 SER649	H-acceptor H-acceptor Pi-H	2.93 3.14 3.62
**4b**	−5.94	4.48 × 10^−5^	−2.8 −0.9 −0.6	ARG651 PHE652 SER649	H-acceptor H-acceptor Pi-H	2.82 3.13 3.62
**4c**	−5.35	1.21 × 10^−4^	−2.8 −0.9	ARG651 PHE652	H-acceptor H-acceptor	2.82 3.13
**4d**	−5.38	1.15 × 10^−4^	−2.8 −0.9	ARG651 PHE652	H-acceptor H-acceptor	2.83 3.13
**4e**	−5.70	6.72 × 10^−5^	−2.8 −0.9	ARG651 PHE652	H-acceptor H-acceptor	2.82 3.13
**4f**	−5.85	5.22 × 10^−5^	−1.7 −2.8 −0.8 −0.6	ASP304 ARG651 PHE652 SER649	H-donor H-acceptor H-acceptor Pi-H	2.93 2.75 3.18 3.60
**4g**	−5.91	4.72 × 10^−5^	−2.1 −2.5 −0.8 −0.6	ARG651 ARG651 PHE652 SER649	H-acceptor H-acceptor H-acceptor Pi-H	2.97 2.69 3.20 3.60
**6**	−6.63	1.4 × 10^−5^	−2.1 −2.9 −0.9	ARG651 ARG651 ASP303	H-acceptor H-acceptor H-acceptor	2.94 3.08 3.03
**8**	−5.60	7.95 × 10^−5^	−2.9 −0.8 −0.6	ARG651 PHE652 SER649	H-acceptor H-acceptor Pi-H	2.77 3.19 3.62
**10**	−5.57	8.36 × 10^−5^	−2.3	ARG651	H-acceptor	2.61
**12**	−5.75	6.17 × 10^−5^	−2.1 −0.6	ARG651 SER649	H-acceptor Pi-H	2.62 3.60
**14a**	−4.52	4.91 × 10^−4^	−0.9	ARG651	Pi-H	3.73
**14b**	−5.08	1.91 × 10^−4^	−2.9 −1.0	CYS301 ARG651	H-donor Pi-H	3.32 3.67
**16**	−4.71	3.56 × 10^−4^	−0.8	ARG651	Pi-H	3.73
**Remdesivir**	−5.48	9.73 × 10^−5^	−1.5 −2.3	ARG651 ARG651	H-acceptor H-acceptor	3.19 3.12

ΔG (Kcal/mol) ^a^; binding free energy.

**Table 2:** Physicochemical properties of the title compounds **1**, **2**, **4a**–**g**, **6**, **8**, **10**, **12**, **14a**, **14b**, and **16**.

Physicochemical Properties										
Compound	Lipophilicity Consensus log P	MW ^a^ g/mol	Heavy Atoms	Aromatic Heavy a Toms	Rot. Bond	HBA ^b^	HBD ^c^	MR ^d^	TPSA ^e^ (A^2^)	% ABS ^f^
**1**	3.33	303.38	21	11	6	4	1	85.45	91.82	77.32
**2**	1.92	289.36	20	11	5	4	3	80.26	120.64	67.38
**4a**	3.80	377.46	27	17	7	4	2	110.53	106.98	71.56
**4b**	3.86	407.49	29	17	8	5	2	117.02	116.21	68.91
**4c**	4.37	411.91	28	17	7	4	2	115.54	106.98	72.09
**4d**	4.46	456.36	28	17	7	4	2	118.23	106.98	72.09
**4e**	3.45	393.46	28	17	7	5	3	112.56	127.21	65.11
**4f**	3.47	393.46	28	17	7	5	3	112.56	127.21	65.11
**4g**	3.87	409.49	29	17	8	5	2	117.02	116.21	68.91
**6**	3.23	367.42	26	16	7	5	2	102.80	120.12	67.56
**8**	ND ^g^	397.52	27	16	7	4	2	113.22	135.22	62.35
**10**	ND	392.48	28	17	7	5	2	113.13	119.87	67.64
**12**	ND	430.53	31	20	7	4	3	127.20	122.77	66.64
**14a**	2.93	355.41	25	16	5	4	2	99.11	120.38	67.47
**14b**	3.91	417.48	30	22	6	4	2	119.58	120.38	67.47
**16**	3.58	353.44	25	16	55	4	1	101.25	100.41	74.36

**MW** ^a^, molecular weight; **HBA** ^b^, number of HB acceptors; **HBD** ^c^, number of HB donors; **MR** ^d^, molar refractivity; **TPSA** ^e^, topological polar surface area; **%ABS** ^f^, percentage of absorption, ND ^g^: not determined.

**Table 3:** Lipinski drug-likeness of compounds **1**, **2**, **4a**–**g**, **6**, **8**, **10**, **12**, **14a**, **14b**, and **16**.

Compound	S *^a^* (mg/L)	Drug-Likeness Model Score	Lipinski Violations	Bioavailability Score
**1**	1.90	0.29	0	0.55
**2**	1.25	−0.01	0	0.55
**4a**	1.50	0.12	0	0.55
**4b**	1.68	0.16	0	0.55
**4c**	5.57	0.65	0	0.55
**4d**	3.96	0.65	0	0.55
**4e**	7.96	0.61	0	0.55
**4f**	7.96	0.28	0	0.55
**4g**	5.42	−0.01	0	0.55
**6**	1.15	0.23	0	0.55
**8**	ND	0.80	0	ND
**10**	ND	0.04	0	ND
**12**	2.93	−0.42	0	0.55
**14a**	1.23	0.47	0	0.55
**14b**	1.20	0.47	0	0.55
**16**	4.04	−0.34	0	0.55

***^a^* S:** solubility.

**Table 4:** List of ADME properties of compounds **1**, **2**, **4a**–**g**, **6**, **8**, **10**, **12**, **14a**,**b**, and **16**.

Compound	Pharmacokinetics					
	Caco-2 ^a^	BBB ^b^	MDCK ^c^	HIA ^d^	PPB ^e^	CYP 2D6 ^f^
**1**	21.25	0.12	10.49	96.60	85.13	Non
**2**	0.46	0.03	9.50	87.95	56.98	Non
**4a**	14.36	0.04	14.23	94.87	91.92	Non
**4b**	16.10	0.03	0.78	95.25	89.70	Non
**4c**	14.38	0.09	0.92	95.15	89.12	Non
**4d**	22.52	0.09	0.03	95.53	87.60	Non
**4e**	1.99	0.08	0.79	92.26	87.62	Non
**4f**	2.47	0.08	2.30	92.26	86.98	Non
**4g**	17.97	0.03	0.716	95.25	88.24	Non
**6**	9.78	0.02	90.64	94.75	88.28	Non
**8**	11.54	0.03	5.08	95.86	86.89	Non
**10**	11.97	0.04	6.69	95.37	82.99	Non
**12**	14.75	0.44	0.27	91.31	88.24	Non
**14a**	3.45	0.12	1.16	94.31	74.99	Non
**14b**	9.85	0.07	0.22	96.19	94.04	Non
**16**	21.68	0.35	0.12	97.47	91.53	Non

**^a^ CACO-2:** permeability through cells derived from human colon adenocarcinoma; **^b^ BBB:** blood-brain barrier penetration; **^c^ MDCK:** permeability through Madin-Darby canine kidney cells; **^d^ HIA:** percentage human intestinal absorption; **^e^ PPB:** plasma protein binding; ^f^ **CYP2D6:** cytochrome P450 2D6.

## Data Availability

The data presented in this study are available on request from corresponding author.

## Supplementary Materials

The following supporting information can be downloaded at: https://www.mdpi.com/article/10.3390/polym14153160/s1, Table S1: Contents. Some selected ^1^H- and ^13^C-NMR spectra of the synthesized compounds and some molecular docking data.

## Abbreviations

RdRP: RNA-dependent RNA; MOE^®^: Molecular Operating Environment; AcOH: Acetic acid; ΔG: the binding free energy; TMS: tetramethylsilane.
