# Supplementary material for: Evaluation of the Binding Relationship of the RdRp Enzyme to Novel Thiazole/Acid Hydrazone Hybrids Obtainable through Green Synthetic Procedure
Source: Polymers (Basel). 2022 Aug 3;14(15):3160. doi: 10.3390/polym14153160 (PMC9371204; doi:10.3390/polym14153160)
Supplement: Supplementary file 1 [file polymers-14-03160-s001.zip › polymers-1789259-supplementary.pdf]

Evaluation of the Binding Relationship of the RdRp Enzyme to Novel Thiazole/Acid Hydrazone Hybrids Obtainable Through Green Synthetic Procedure

Jehan Y. Al-Humaidi[1], Mohamed G. Badrey[2,3], Ashraf A. Aly[4], AbdElAziz A. Nayl[5,*], Mohie E. M. Zayed[6], Ohoud A. Jefri[6] and Sobhi M. Gomha[7,8*]

[1] Department of Chemistry, College of Science, Princess Nourah bint Abdulrahman University, P.O .BOX 84428, Riyadh 11671, Saudi Arabia, jyalhamidi@pnu.edu.sa.
[2] Chemistry Department; Faculty of Science; Fayoum University, El-Fayoum, Egypt; mgb00@fayoum.edu.eg.
[3] Chemistry Department; Faculty of Science and Arts-Almandaq; Al-Baha University, Al-Baha, Saudia Arabia
[4] Chemistry Department, Faculty of Science, Organic Division, Minia University, 61519-El-Minia, Egypt, ashrafaly63@yahoo.com, ashraf.shehata@mu.edu.eg.
[5] Department of Chemistry, College of Science, Jouf University, Sakaka 72341, Aljouf, Saudi Arabia. aa-nayel@ju.edu.sa, aanayl@yahoo.com
[6] Department of Chemistry, Faculty of Science, King Abdulaziz University, Jeddah 21589, Saudi Arabia, MZAYED@kau.edu.sa (MEMZ), jeefrio0@gmail.com (OAJ)
[7] Department of Chemistry, Faculty of Science, Cairo University, Giza, 12613, Egypt, s.m.gomha@cu.edu.eg
[8] Department of Chemistry, Faculty of Science, Islamic University of Madinah, Madinah, 42351, Saudi Arabia. smgomha@iu.edu.sa
*Correspondence: aanayel@ju.edu.sa or aanayl@yahoo.com (A.A.N.); s.m.gomha@cu.edu.eg or smgomha@iu.edu.sa (S.M.G.)

Table S1: Contents

No.	Spectra	No.	Spectra
1	Table S1	10	^{1}H-NMR spectrum of compound **4g**
2	^{1}H-NMR spectrum of compound **1**	11	^{13}C-NMR spectrum of compound **4g**
3	^{1}H-NMR spectrum of compound **4a**	12	^{1}H-NMR spectrum of compound **6**
4	^{1}H-NMR spectrum of compound **4b**	13	^{1}H-NMR spectrum of compound **10**
5	^{13}C-NMR spectrum of compound **4b**	14	^{1}H-NMR spectrum of compound **12**
6	^{1}H-NMR spectrum of compound **4c**	15	^{13}C-NMR spectrum of compound **12**
7	^{1}H-NMR spectrum of compound **4d**	16	^{13}C-NMR spectrum of compound **14a**
8	^{1}H-NMR spectrum of compound **4e**	17	^{1}H-NMR spectrum of compound **14b**
9	^{13}C-NMR spectrum of compound **4e**		

2.3.Physicochemical properties, pharmacokinetics, and drug-likeness profile; in silico Prediction [35-40]

Recently, The most promising compounds can be picked in silico ADMET screens, reducing the chance of degradation of drugs in late stages. To achieve a desired *in vivo* goal, it should be balance between pharmacodynamics and pharmacokinetic properties. Also, Further information about regimen and drug dose are given by the prediction of brain penetration, volume of distribution, oral bioavailability, and clearance. Many parameters such as drug solubility S, partition coefficients, polar surface PSA, cell permeability, human intestinal absorption HIA, and drug-likeness score have been studied during virtual screening methods. An available orally drug elected in agreement with Lipinski's rule If the molecular weight is less than 500, Log P is not higher than 5, the number of hydrogen bond acceptors is less than 10 and the number of donor hydrogen bond donors is less than 5. The number of rotatable bonds reflects molecular flexibility that plays an important role in oral bioavailability and means less orally active in a flexible molecule. The number of hydrogen bonding groups has also been suggested as a consideration to substitute for the polar surface area (PSA) and also to measure the percentage absorption (%ABS) as it is in inversely proportional to tPSA %ABS = 109 − 0.345 tPSA.

The higher oral bioavailability exhibited by Compounds with tPSA of less than 140 A2 and 10 or fewer rotatable bonds. Herein, we used Molinspiration, Molsoft, and SwissADME software for predicting the pharmacokinetic parameters of the reported compounds.

In addition, the following pharmacokinetic parameters have been tried in silico using Pre-ADMET software: blood-brain barrier partitioning coefficient (BBB), cytochrome inhibition of cytochrome P4502D6 (CYP2D6), MDCK (Madden), Caco2, parameter (human colon cancer). MDCK (Madin–Darby canine kidney cells) coefficient of permeability, human intestinal absorption (HIA) and human plasma protein binding (PPB).

3.2 Docking study

The docking simulation investigation was performed using Molecular Operating Environment (MOE®) version 2014.09, Chemical Computing Group Inc., The computational software operated under “Windows XP” installed on an Intel Pentium IV PC with a 1.6 GHz processor and 512 MB of memory. The target compounds were generated in a 3D model using the MOE builder interface. After examining their structures and formal charges on the atoms by means of 2D depiction, the following steps were carried out:

- All conformers underwent energy minimization, all minimizations were performed with MOE up to RMSD gradient of 0.01 kcal/mol and RMS distance (root mean square) 0.1 Å with MMFF94X force field and partial charges were calculated automatically.

- The obtained database was then saved as a Molecular Database (MDB) file for use in the docking calculations.

3.3. Target optimization:

X-ray crystal structure of the target enzyme Topoisomerase II (PDB: 7bv2) obtained from the Protein Data Bank. The compounds were docked at the active site of the target enzyme.

The enzyme was prepared for docking studies by:

• The co-crystallized ligand has been omitted.

• Hydrogen atoms have been added to the system with their standard geometry.

• The connection and type of atoms are checked for any errors with automatic correction.

• The choice of the receptor and its atoms potential were fixed.

3.4. Docking of the target molecules to SARS-Cov-2 RNA-Dependent RNA (RdRP) active site

The target compounds were docked using the MOE-Dock software. The following methodology has generally been applied:

• The file of enzyme active site has been loaded, and the Dock is started. The program specification has been modified to:

- Dummy atoms as the docking site.
- Triangle matcher as the placement methodology to be used.
- London dG as a scoring methodology to use and adjust to their default values.

• The MDB file for the ligand was loaded to be docked has been loaded and Dock calculations were run automatically

The obtained poses were studied, and the obtained poses showed that the best ligand enzyme interactions were selected and stored for energy calculations.

3.5. Physicochemical properties, pharmacokinetics, and drug-likeness profile; in silico Prediction

The lipid and physicochemical profiles of the target compounds were collected using the and Molinspiration software (https://standingmet.bmdrc.kr/) and Swiss ADME software (http://www.swissdme.ch/index.php). In addition, drug-likeness

to the target compounds was obtained using Swiss ADME software and Molsoft (https://www.molsoft.com/). The absorption percentage was calculated by this equation:

$$\%ABS = 109 - 0.345\ tPSA$$

where TPSA: topological polar surface area; % ABS: Absorption percentage.

References

35. Daina, A.; Michielin, O.; Zoete, V. SwissADME: a free web tool to evaluate pharmacokinetics, drug-likeness and medicinal chemistry friendliness of small molecules. *Sci. Rep.* **2017**, *7*, 42717.

36. De, H. V.; Waterbeemd, E. Gifford, ADMET *in silico* modelling: towards prediction paradise? *Nature reviews Drug discovery* **2003**, *2*, 192-204.

37. Veber, D. F.; Johnson, S. R.; Cheng, H.-Y.; Smith, B. R.; Ward, K.W.; Kopple, K. D. Molecular properties that influence the oral bioavailability of drug candidates. *J. Med. Chem.* **2002**, *45*, 2615-2623.

38. Rajanarendar, E.; Krishna, S. R.; Nagaraju, D.; Reddy, K. G.; Kishore, B.; Reddy, Y. Environmentally benign synthesis, molecular properties prediction and anti-inflammatory activity of novel isoxazolo [5,4-d]isoxazol-3-yl-aryl-methanones via vinylogous Henry nitroaldol adducts as synthons. *Bioorg. Med. Chem. Lett.* **2015**, *25*, 1630-1634.

39. Wetzel, S.; Schuffenhauer, A.; Roggo, S.; Ertl, P.; Waldmann, H. Cheminformatic analysis of natural products and their chemical space. *CHIMIA Inter. J. Chem.* **2007**, *61*, 355-360.

40. Arnautova, Y. A.; Abagyan, R. A.; Totrov, M. Development of a new physics-based internal coordinate mechanics force field and its application to protein loop modeling. *Proteins* **2011**, *79*, 477-498.

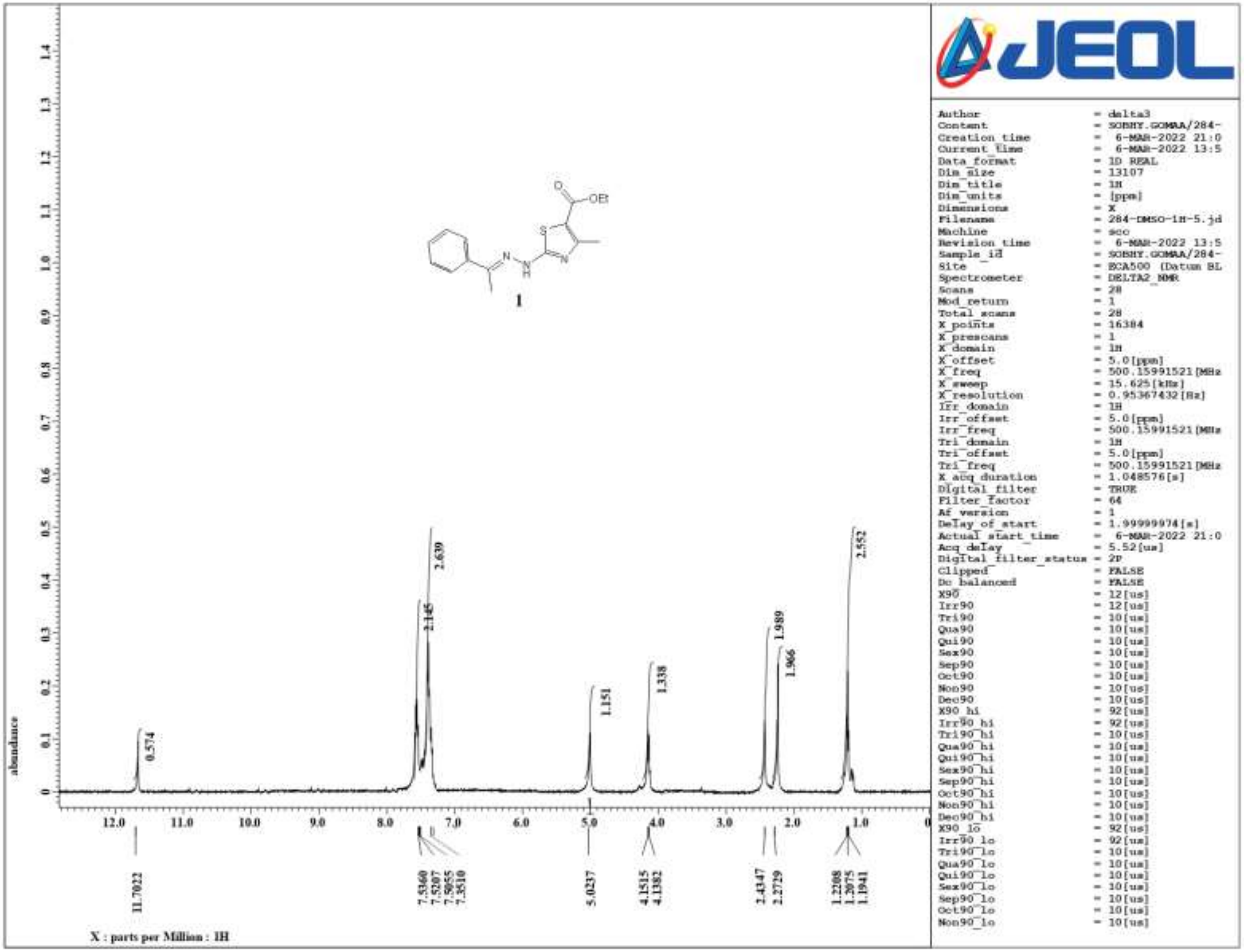

^{1}H-NMR spectrum of compound 1

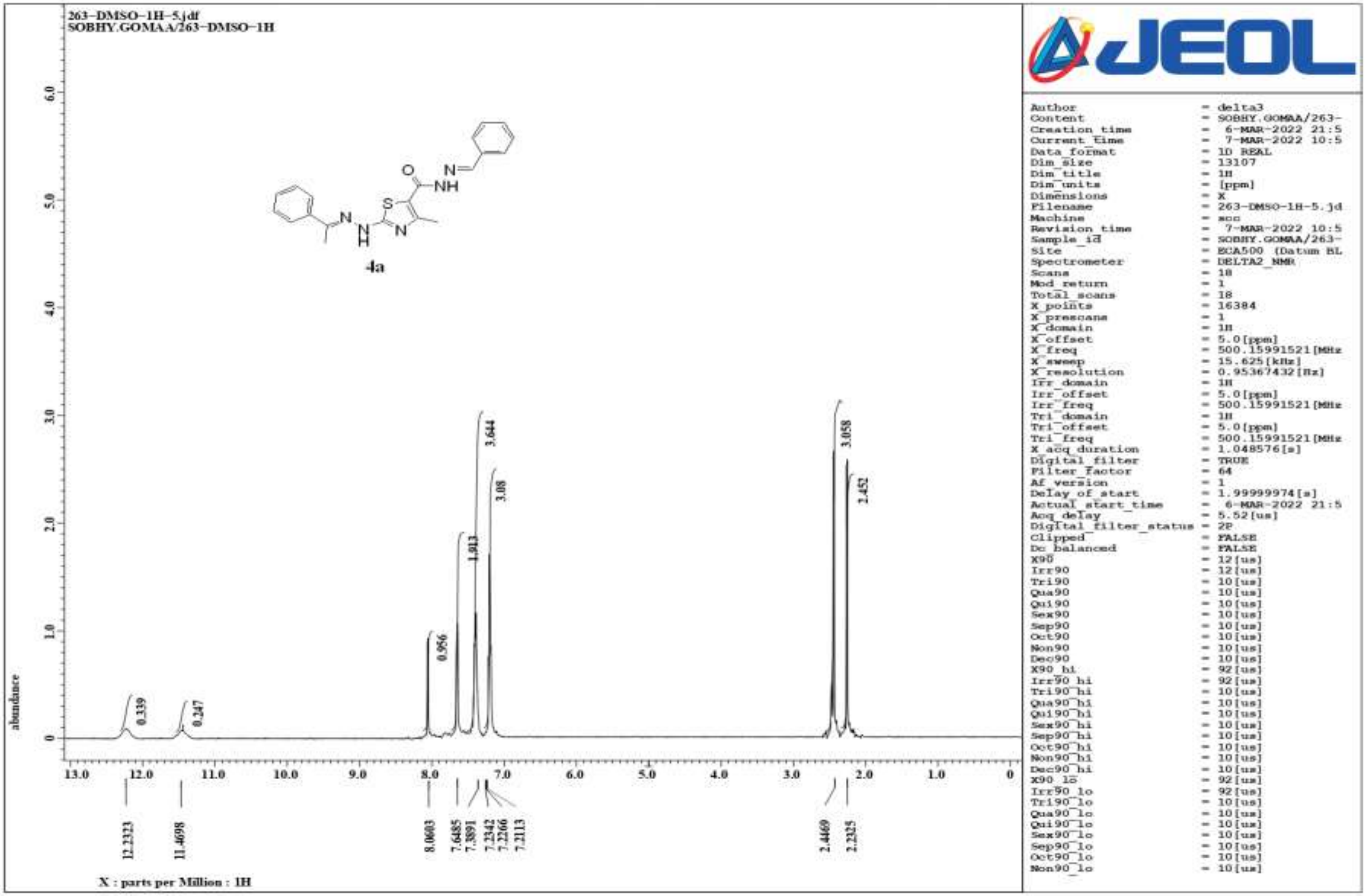

^{1}H-NMR spectrum of compound 4a

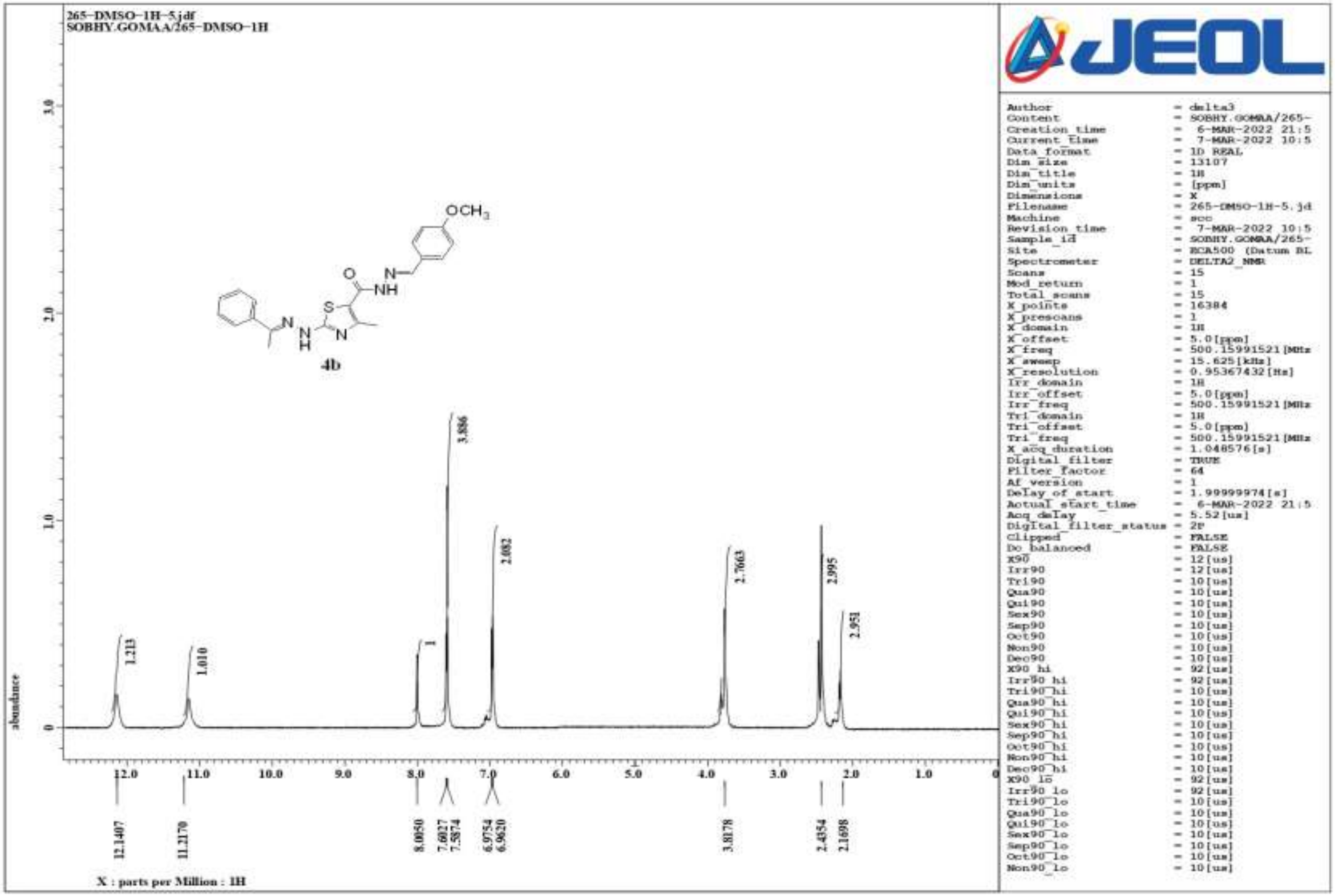

^{1}H-NMR spectrum of compound 4b

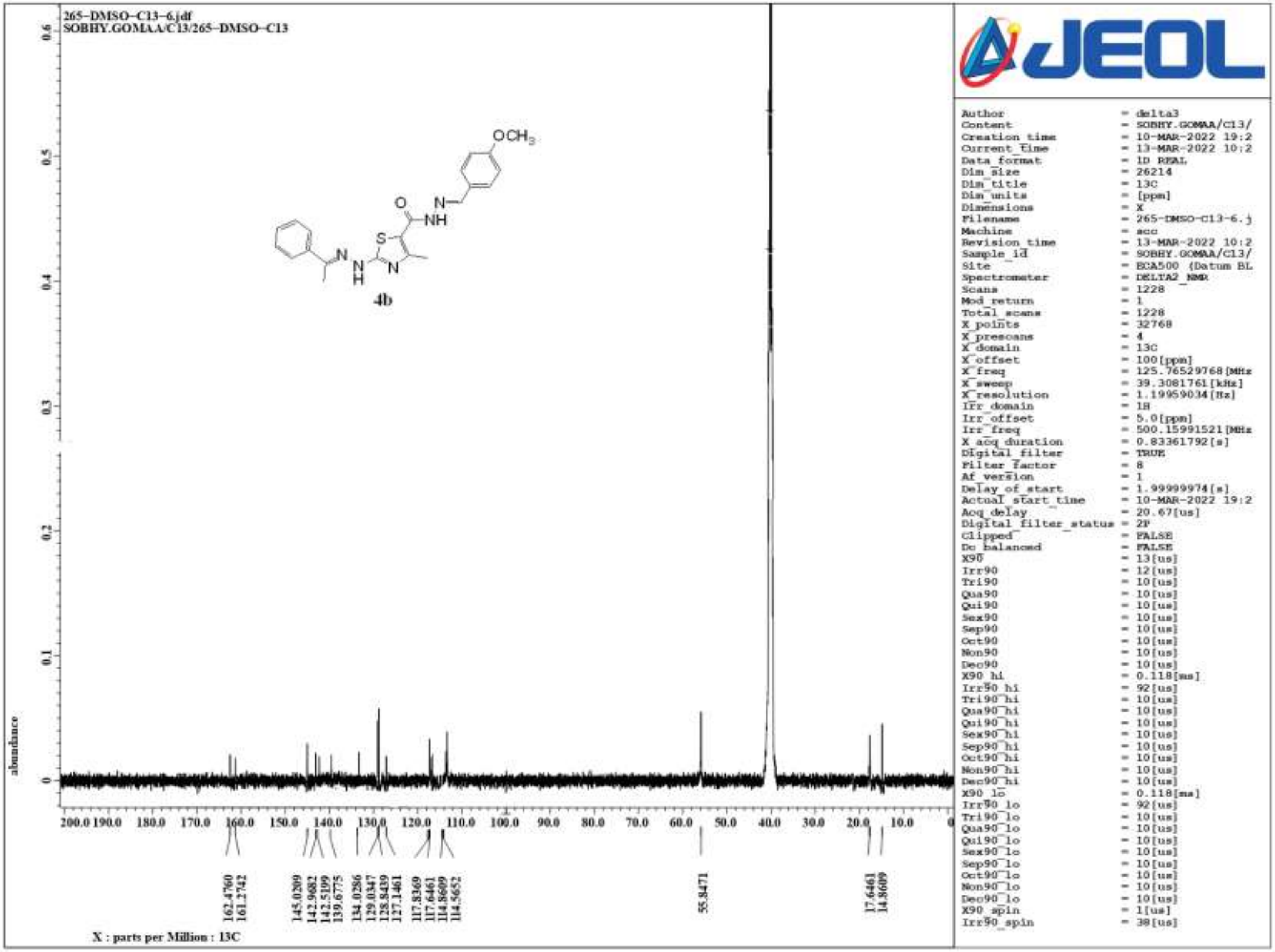

^{13}C-NMR spectrum of compound 4b

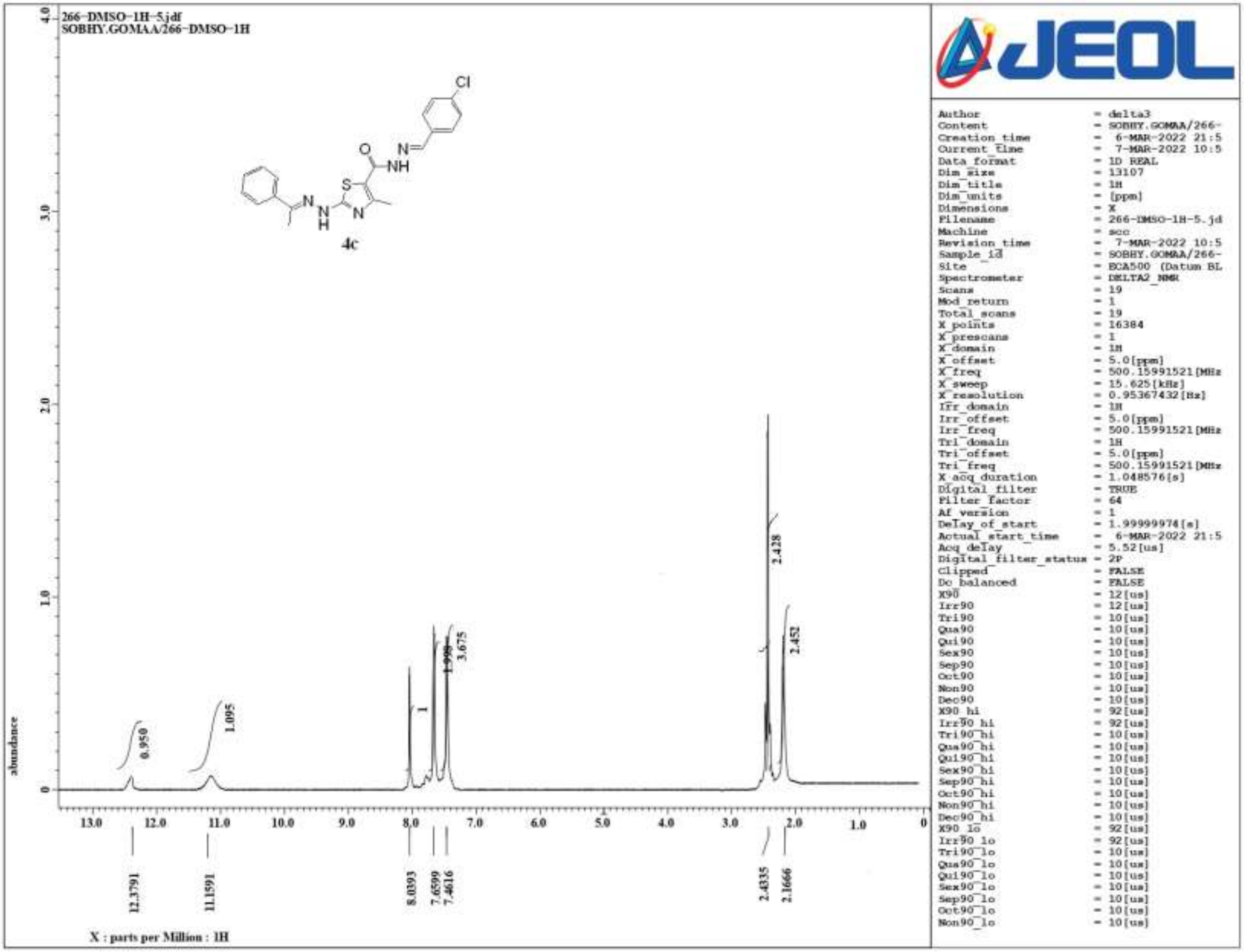

^{1}H-NMR spectrum of compound 4c

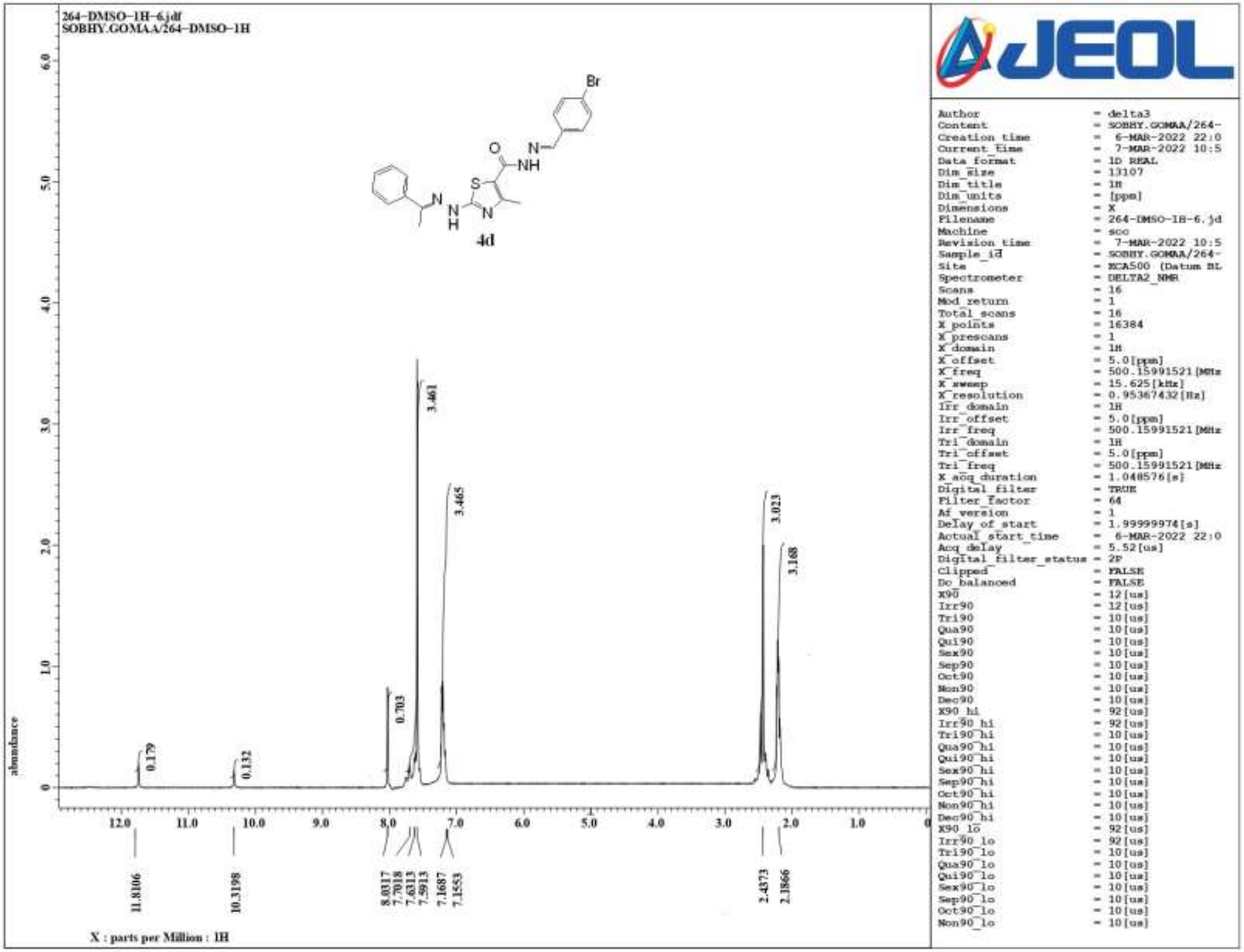

^{1}H-NMR spectrum of compound 4d

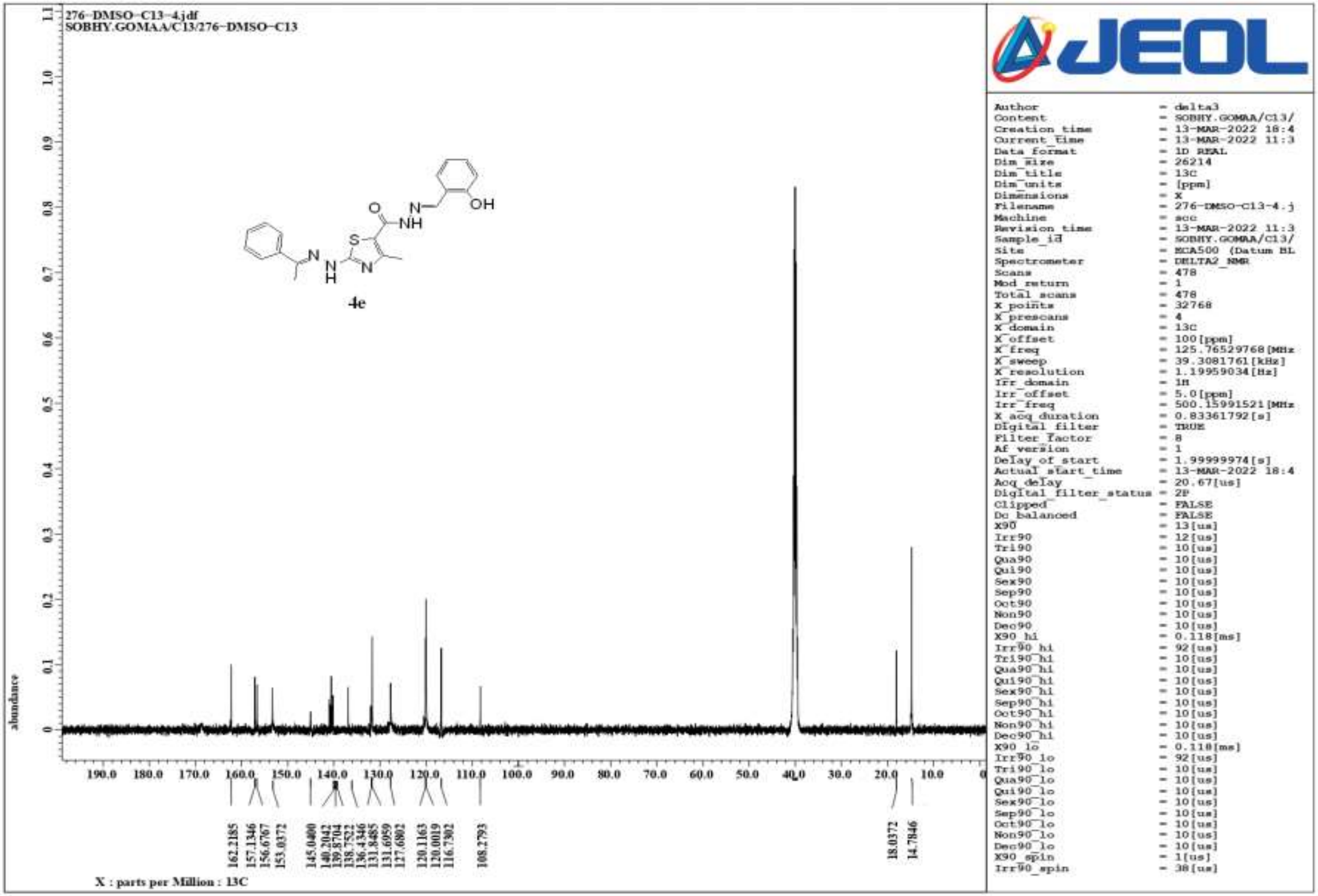

^{13}C-NMR spectrum of compound 4e

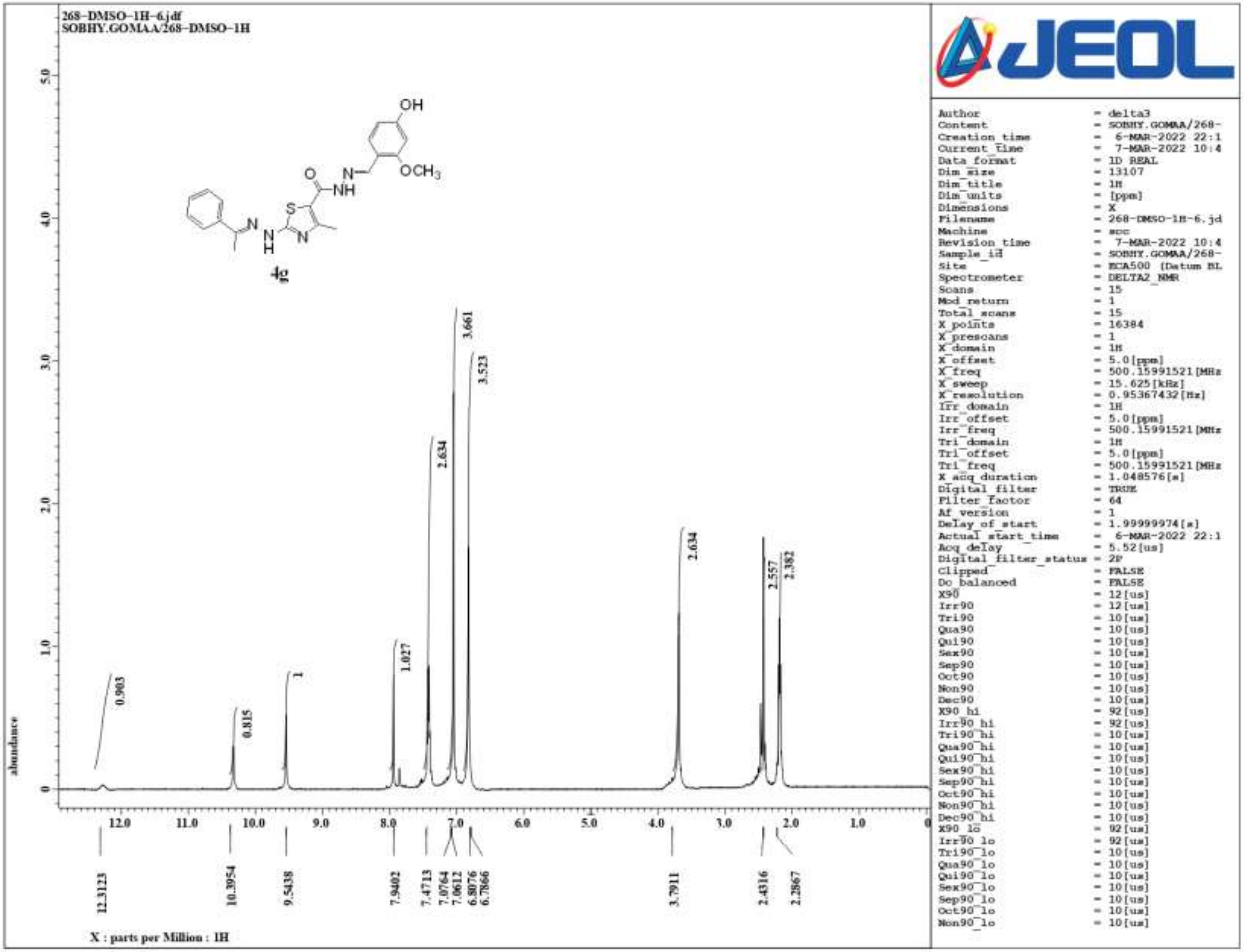

^{1}H-NMR spectrum of compound 4g

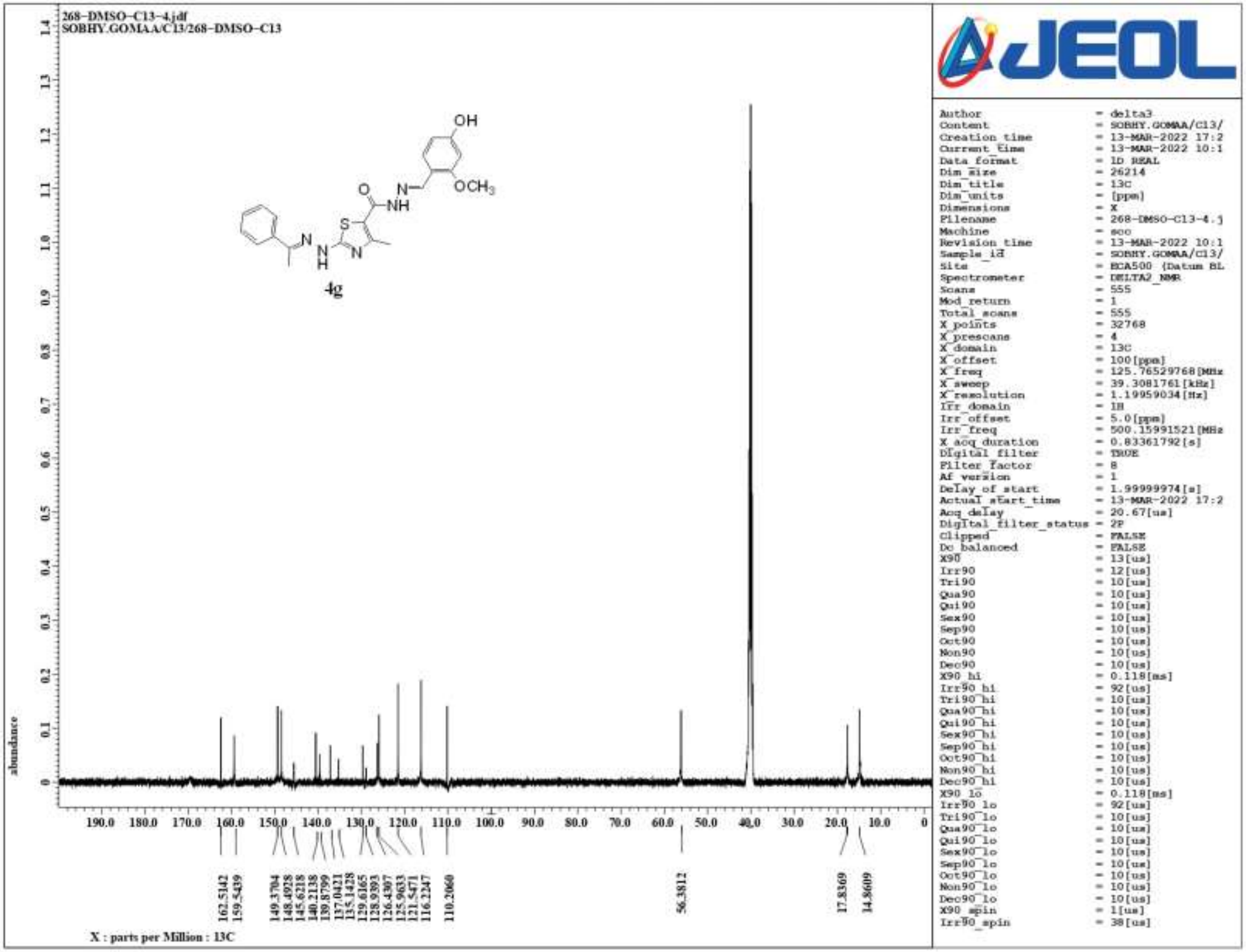

^{13}C-NMR spectrum of compound 4g

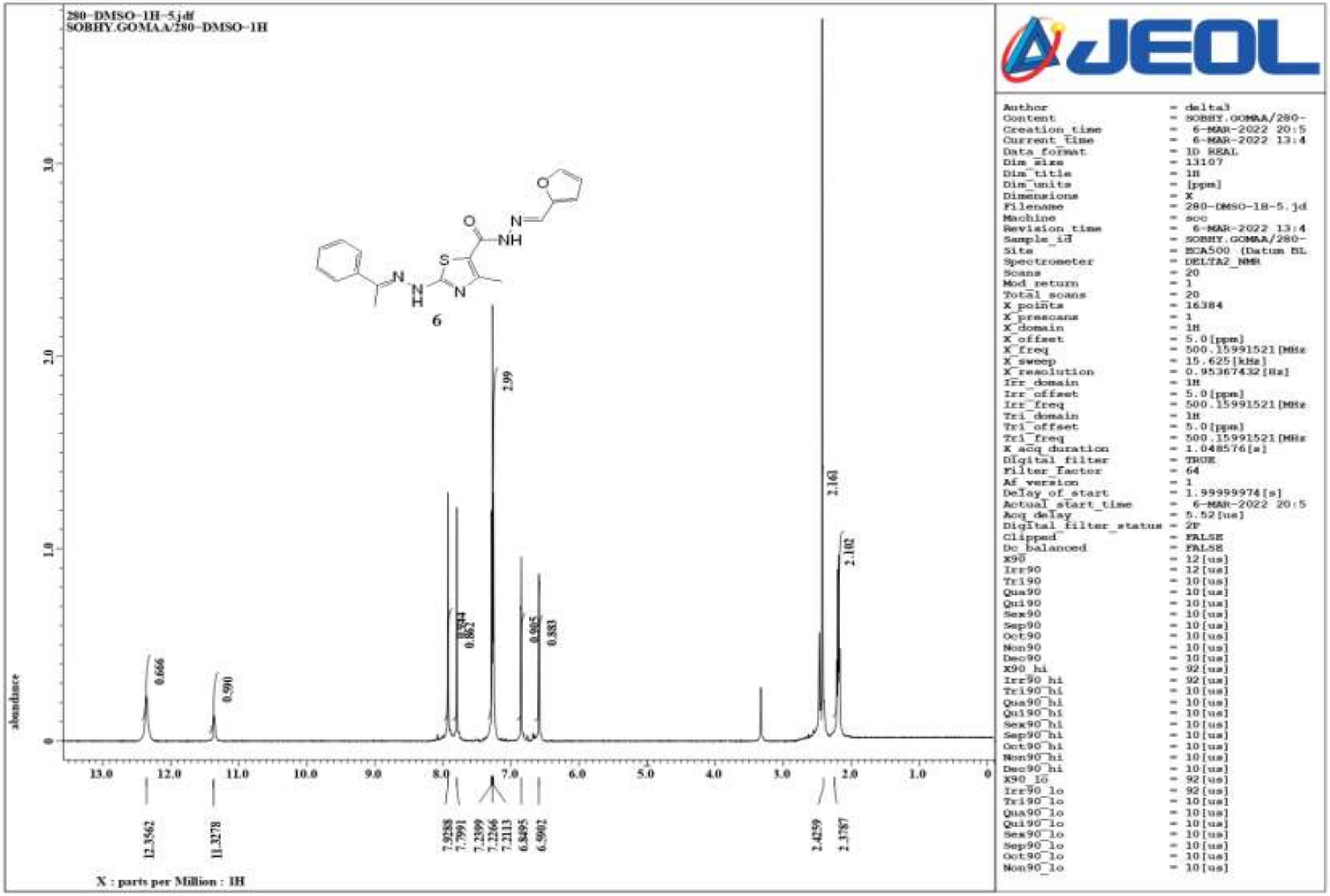

^{1}H-NMR spectrum of compound 6

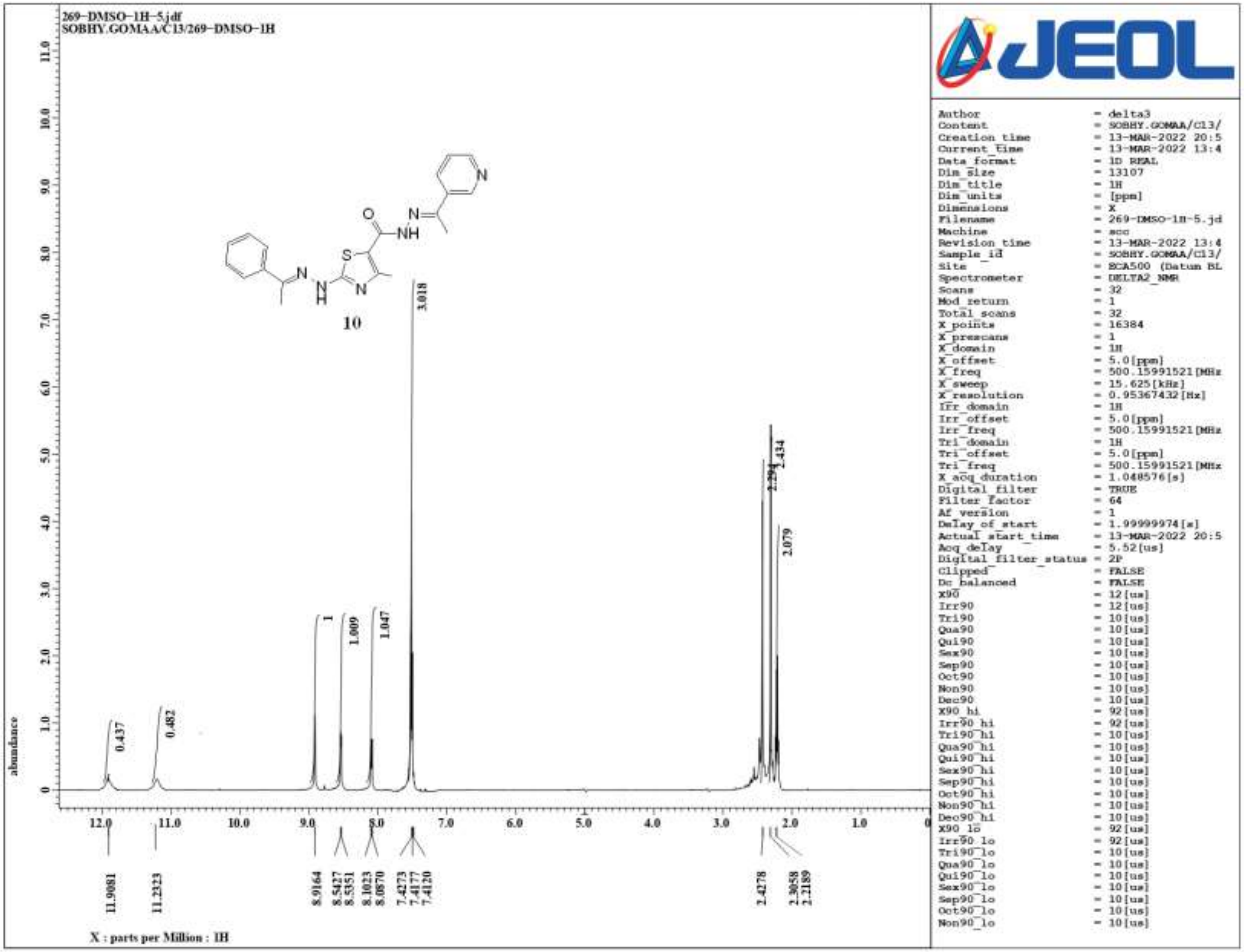

^{1}H-NMR spectrum of compound 10

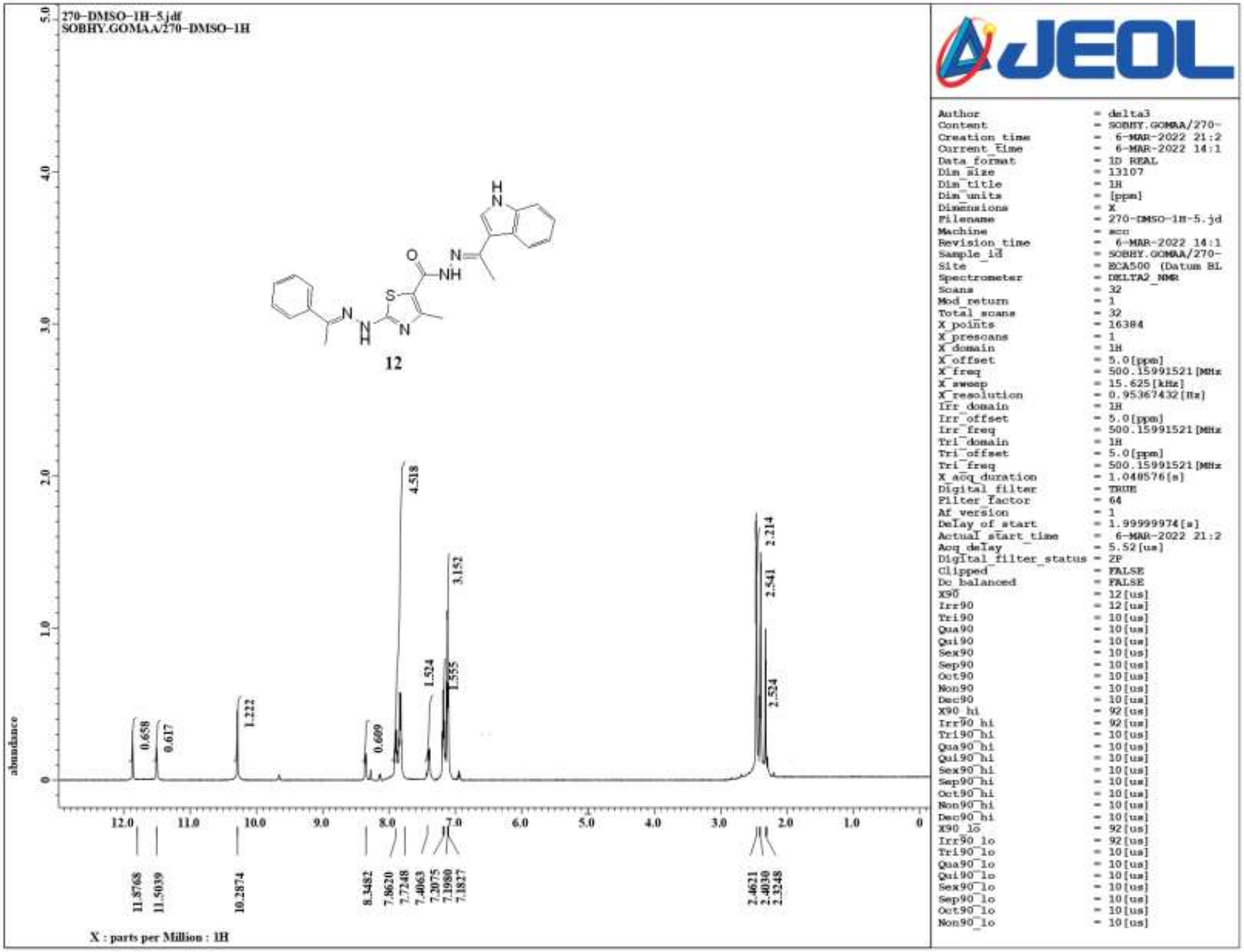

^{1}H-NMR spectrum of compound 12

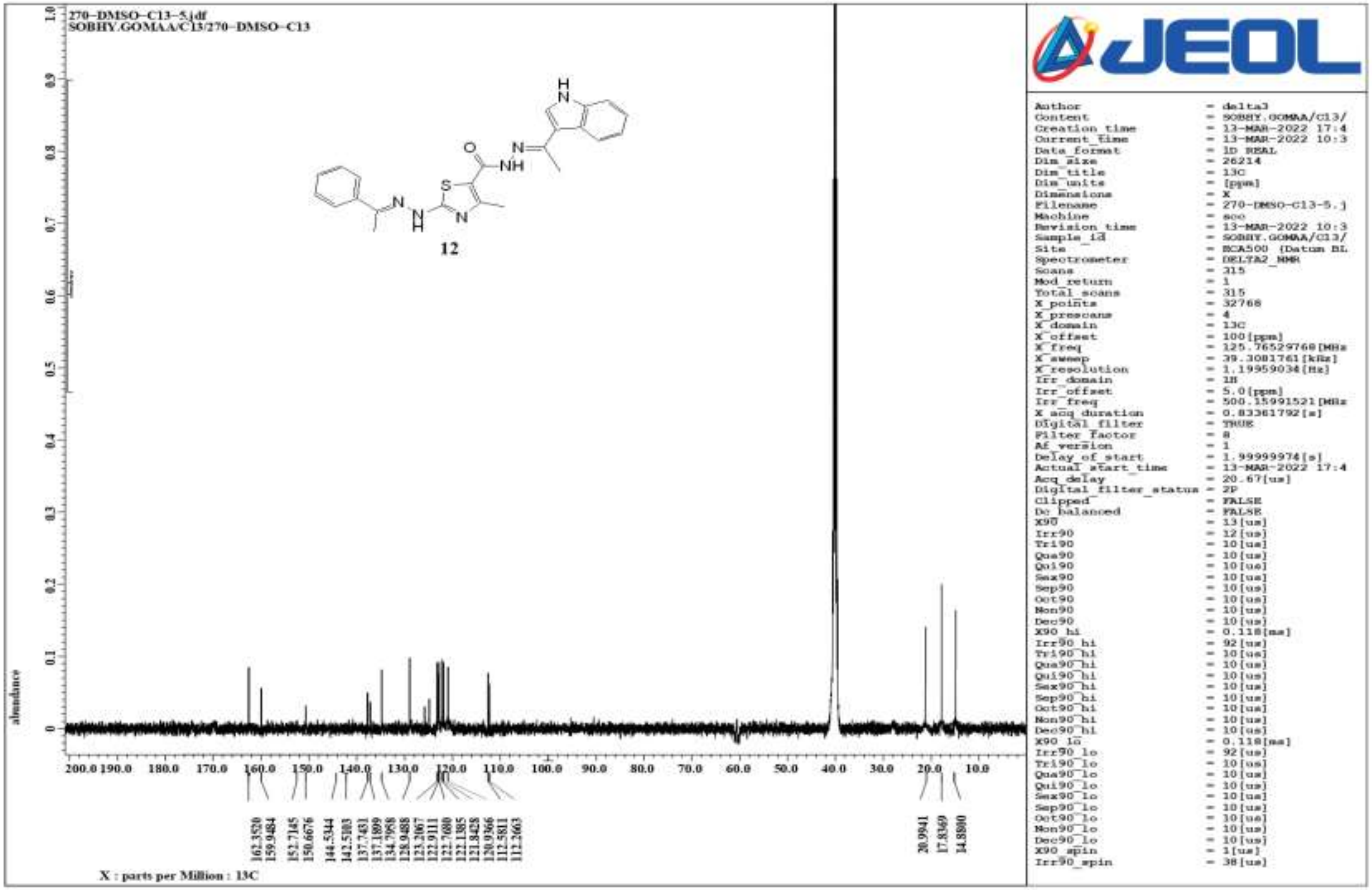

^{13}C-NMR spectrum of compound 12

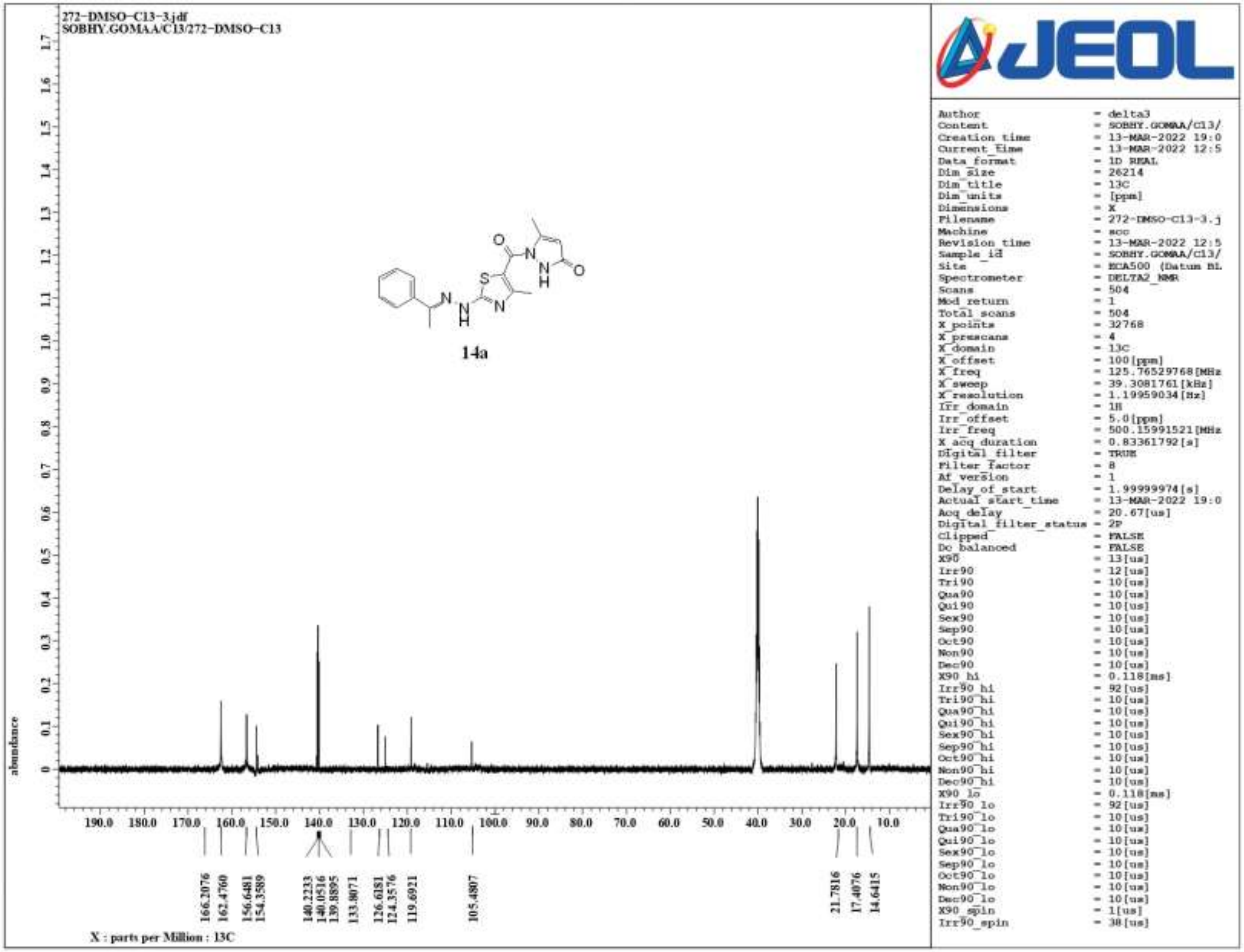

^{13}C-NMR spectrum of compound 14a

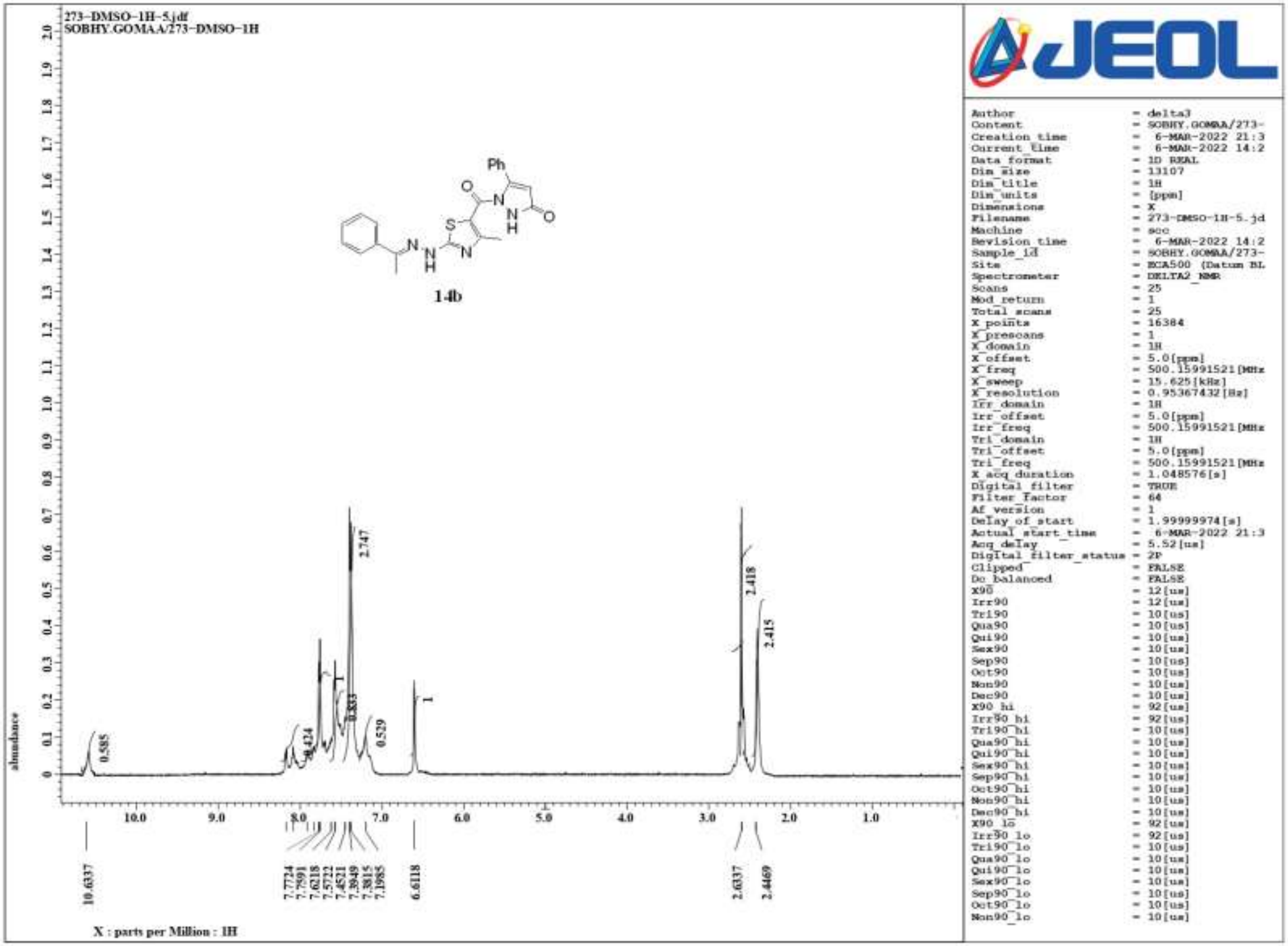

^{1}H-NMR spectrum of compound 14b
